# GenomeDecoder: inferring segmental duplications in highly repetitive genomic regions

**DOI:** 10.1093/bioinformatics/btaf058

**Published:** 2025-02-05

**Authors:** Zhenmiao Zhang, Ishaan Gupta, Pavel A Pevzner

**Affiliations:** Department of Computer Science and Engineering, University of California San Diego, La Jolla, CA 92093, United States; Department of Computer Science, Hong Kong Baptist University, Hong Kong SAR, China; Department of Computer Science and Engineering, University of California San Diego, La Jolla, CA 92093, United States; Department of Computer Science and Engineering, University of California San Diego, La Jolla, CA 92093, United States

## Abstract

**Motivation:**

The emergence of the ‘telomere-to-telomere’ genomics brought the challenge of identifying segmental duplications (SDs) in complete genomes. It further opened a possibility for identifying the differences in SDs across individual human genomes and studying the SD evolution. These newly emerged challenges require algorithms for reconstructing SDs in the most complex genomic regions that evaded all previous attempts to analyze their architecture, such as rapidly evolving immunoglobulin loci.

**Results:**

We describe the GenomeDecoder algorithm for inferring SDs and apply it to analyzing genomic architectures of various loci in primate genomes. Our analysis revealed that multiple duplications/deletions led to a rapid birth/death of immunoglobulin genes within the human population and large changes in genomic architecture of immunoglobulin loci across primate genomes. Comparison of immunoglobulin loci across primate genomes suggests that they are subjected to diversifying selection.

**Availability and implementation:**

GenomeDecoder is available at https://github.com/ZhangZhenmiao/GenomeDecoder. The software version and test data used in this paper are uploaded to https://doi.org/10.5281/zenodo.14753844.

## 1 Introduction

**Figure 1.**
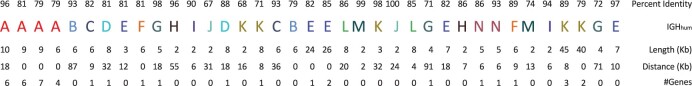
The block decomposition of the human IGH locus. The IGH locus in the reference human genome hg38 (denoted as IGH_hum_) is formed by 37 block-instances representing 14 distinct repeated blocks (duplication subunits) of multiplicity varying from 2 to 5 (denoted by letters from A to N). The 37 block-instances are separated by 36 non-repetitive segments that are not shown (some of these segments have length 0). The row above the block decomposition shows the percent identity between each block-instance in IGH_hum_ against the consensus sequence of the block. The three rows below the block decomposition provide information about the distances between consecutive block-instances, the length of each block-instance, and the number of immunoglobulin (V, D, or J) genes in each block-instance. The number of genes is defined based on the annotated hg38 human genome reference. In total, 18 out of 48 annotated V genes, 23 out 27 annotated D genes, and 0 out 6 annotated J genes occur in duplication subunits (the remaining genes occur in non-repetitive regions). All 23 D genes in the duplication subunits occur in the four-unit tandem repeat AAAA.

Since each genome has undergone duplications, deletions, and rearrangements, decoding genome architectures (inference of *synteny blocks* (*SBs*), *segmental duplications* (*SDs*), and *tandem duplications* (*TDs*)) is a challenging problem. For example, mammalian immunoglobulin (IG) loci, shaped by SDs and TDs, have widely variable architectures even within the human population (Rodriguez *et al.* 2023), let alone between different primate genomes. As a result, decomposing them into *duplication subunits* (Jiang *et al.* 2007) is a prerequisite for both biomedical studies of IG loci across the human population and their comparative genomics studies across mammals. Understanding how structural variations (SVs) in IG loci affect variations in the antibody repertoires is critical for personalized immunogenomics and vaccine design (Watson *et al.* 2017). However, decomposing the human IG architecture into duplication subunits remained an open problem until recently. We illustrate the complex architecture of human immunoglobulin heavy chain (IGH) locus by decomposing it into duplication subunits (Fig. 1). For brevity, since the problems of inferring SBs, SDs, and TDs are similar to each other, below we refer to both SBs and units of SDs/TDs simply as *blocks*.

Shortly after the first mammalian genomes had been sequenced, Pevzner and Tesler (2003) developed the GRIMM-Synteny algorithm for generating non-overlapping blocks and Kent *et al.* (2003) described the ‘chains-and nets’ algorithm for generating potentially overlapping blocks. The key advantage of the non-overlapping representation is that it enables compact representation of genomic architectures in the alphabet of all blocks (Fig. 1), a prerequisite for downstream comparative genomics and evolutionary studies such as analysis of genome rearrangements (Bourque *et al.* 2004, Peng *et al.* 2006).

We emphasize that different papers often use different and informal concepts of the block and do not explicitly define the objective function of the block generation. This may lead to ill-defined blocks and incorrect biological conclusions as illustrated by a controversy discussed by Peng *et al.* (2006) and Sankoff (2006).

Even though it is difficult to compare various approaches to block generation, most of them use the concept of a block that is similar to the one defined in the GRIMM-Synteny algorithm (Pevzner and Tesler 2003). However, GRIMM-Synteny and other early block inference algorithms (Darling *et al.* 2004, Haas *et al.* 2004, Ma *et al.* 2006, Grabherr *et al.* 2010, Drillon *et al.* 2014) did not properly address inference of blocks in the case of extensive duplications and deletions, e.g. they were not able to infer the structure of SDs in a mammalian genome, infer blocks in highly repetitive regions (*HRRs*) such as IG loci, or infer blocks in genomes that have undergone whole-genome duplications (WGDs). Liu *et al.* (2018) benchmarked various block generation algorithms and commented that they agree on long megabase-size blocks but face challenges in analyzing ‘local shuffles.’

The problem of decomposing the genome into the alphabet of non-overlapping blocks was first addressed by Jiang *et al.* (2007) who revealed the mosaic structure of SDs in the human genome. Paten *et al.* (2008) and Pham and Pevzner (2010) further described Enredo and DRIMM-Synteny algorithms for inferring blocks in highly duplicated genomes. Minkin *et al.* (2013) improved on DRIMM-Synteny by developing the Sibelia algorithm, while Krasheninnikova *et al.* (2020) improved on Enredo by developing the halSynteny algorithm. Recently, Pu *et al.* (2018) and Iseric *et al.* (2022) developed SDquest and BISER algorithms for identifying ancient SDs.

The problem of inferring blocks is similar to the problem of *de novo* repeat classification that was addressed using the concept of the *A-Bruijn graph* (Pevzner *et al.* 2004). Pham and Pevzner (2010) modified the A-Bruijn graph approach for block generation in highly duplicated genomes such as yeast genomes subjected to WGD. The key ingredient of their approach is the *graph simplification algorithm* that collapses bubbles in the A-Bruijn graph of a genome. However, the human genome contains many HRRs (even more complex than the entire yeast genomes that have undergone the WGDs) that are not adequately represented by simple bubbles in the A-Bruijn graph. Moreover, DRIMM-Synteny first represents a genome in the alphabet of genes (with similar genes represented by the same ID) and transforms this representation into blocks. This approach has limitations since many genomic regions contain few genes and since establishing similarity between some genes (e.g. short and rapidly evolving D genes in the IG loci) is challenging. Of course, one can substitute ‘similar genes’ by ‘shared *k*-mers’ and apply a block reconstruction algorithm to genomes represented in the alphabet of shared *k*-mers instead of similar genes. However, any fixed choice of the *k*-mer size would either lead to spurious similarities (when *k* is small) or to undetected similarities (when *k* is large). The Sibelia algorithm (Minkin *et al.* 2013) addressed this limitation of DRIMM-Synteny by incorporating its graph simplification algorithm into the *iterative de Bruijn graph* framework that performs graph simplifications across the de Bruijn graphs (*DBGs*) with progressively increasing *k*-mer sizes.

We applied halSynteny (Krasheninnikova *et al.* 2020), Sibelia (Minkin *et al.* 2013), and SDquest (Pu *et al.* 2018) to analyze some of the most complex regions in primate genomes, revealed some limitations of these tools, and developed the GenomeDecoder algorithm to address these limitations. GenomeDecoder borrows the graph simplification idea from DRIMM-Synteny and the iterative DBG idea from Sibelia.

**Figure 2.**
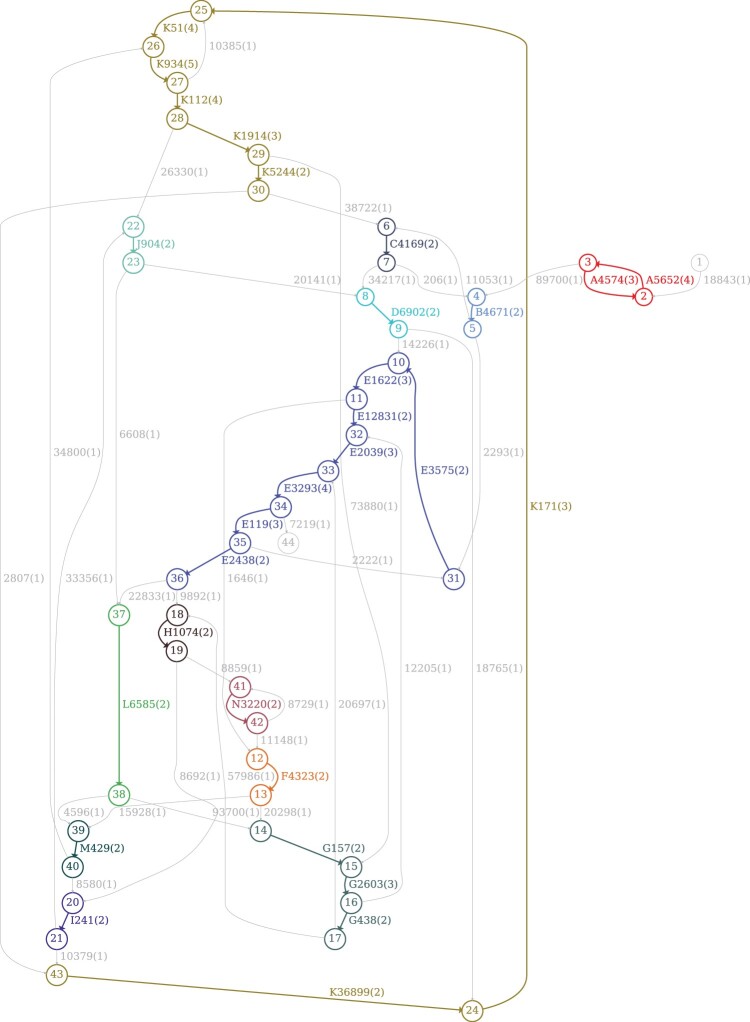
The de Bruijn graph of the disembroiled human IGH locus. The de Bruijn graph of the disembroiled IGH locus IGH_hum_ for the *k*-mer size 2000. Each vertex is represented as a circle labeled by the vertex IDs. The edge label consists of the block ID in Fig. 1 (if any), the length of the edge and its multiplicity (in the parenthesis). The labels of the duplicated blocks are boldfaced. The figure is generated by the GraphViz tool (version 2.43.0).

If blocks represented exact repeats, the block decomposition problem would be easy since the DBG on *k*-mers provides a comprehensive representation of all *exact* repeats of length at least *k* in a genome (each edge represents a block). In practice, analysis of blocks faces the challenge of compactly representing the mosaic structure of all inexact repeats in a genome. Similar to Sibelia, GenomeDecoder iteratively modifies (*disembroils*) the genome by transforming its inexact repeats into the exact ones using a series of the DBG simplification operations (that both DRIMM-Synteny and Sibelia use) with iteratively increasing *k*-mer sizes. The DBG of the disembroiled genome enables block inference because it is greatly simplified as compared to the DBG of the original genome. Figure 2 presents the DBG of the disembroiled human IGH locus that enabled inference of blocks shown in Fig. 1. An important contribution of GenomeDecoder (as compared to DRIMM-Synteny, Sibelia, and the algorithm by Jiang *et al.* 2007) is a more extensive set of graph simplifications that allows it to infer the blocks even in the most complex genomic regions.

Even though block inference is a prerequisite for downstream comparative genomics analysis, the previously published papers on block inference stopped short of the downstream applications. Here, we illustrate how block decompositions generated by GenomeDecoder shed light on important questions in comparative genomics. For example, predicting genes and revealing orthologous/paralogous mammalian genes in the rapidly evolving IG loci is a challenging task, particularly in the case of short D genes (Sirupurapu *et al.* 2022). Difficulties in establishing orthologous/paralogous relationships between genes make it difficult to analyze the mutation rates and the extent of selective pressures in these loci. We illustrate how block decompositions lead to predicting novel D genes and analysis of the selective pressure on these genes.

## 2 Materials and methods

### 2.1 Outline of the GenomeDecoder algorithm

We first describe how GenomeDecoder works for a single genome represented as a set of strings (each string encodes a chromosome) and later generalize this algorithm for multiple genomes. GenomeDecoder (i) transforms the original genome into the disembroiled genome where imperfect repeats are transformed into identical repeats, (ii) constructs the DBG of the disembroiled genome and infers blocks from this DBG, and (iii) generates blocks in the original genome by aligning them against blocks in the disembroiled genome.

To decompose a string-set *Genome* into blocks. GenomeDecoder selects an initial (small) *k*-mer size and uses the LJA assembler (Bankevich *et al.* 2022) to construct the *condensed de Bruijn graph* DB_*k*_(*Genome*) on the set of all *k*-mers in *Genome* (edge-weights in this graph represent multiplicities of *k*-mers in *Genome*). The condensed DBG is a compact representation of the DBG where each non-branching path is substituted by a single edge. For brevity, we will refer to the condensed DBG simply as the DBG.

**Figure 3.**
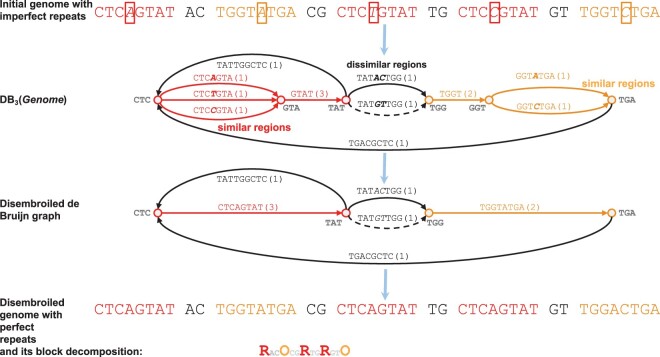
Outline of a single iteration of the GenomeDecoder algorithm. The input is a ‘genome’ with three instances of the repeat CTC‘X’GTAT and two instances of the TGGT‘X’TGA repeat, where ‘X’ denotes substitutions. The graph DB_3_(*Genome*) reveals three bubbles that are used to identify similar (the leftmost and the rightmost bubbles with a single substitution) and dissimilar (the middle bubble with two substitutions) regions. GenomeDecoder transforms the initial DBG into a disembroiled graph by collapsing bubbles formed by similar regions. The traversal of the initial graph translates into a traversal of the transformed graph that reveals a disembroiled genome’. Edges of the transformed DBG correspond to blocks. There are two repetitive blocks in the transformed genome (CTCAGTAT (R) and TGGTATGA (O) blocks) that were ‘hidden’ in the initial DBG. The traversal of this graph reveals the decomposition **R**_AC_**O**_CG_**R**_TG_**R**_GT_**O** of the disembroiled genome into blocks (_AC_, _CG_, _TG_, and _GT_ represent non-repetitive regions). After generating blocks in the disembroiled genome, GenomeDecoder aligns it against the original genome to generate the blocks in the original genome (this step is not shown). Although this example only shows simple bubbles, GenomeDecoder analyzes complex bubbles as well.

A genome formed by *n* strings (chromosomes) represents a *traversal* of its DBG by *n* paths that visit each edge of the graph at least once. GenomeDecoder starts from a small *k*-mer-size and uses DB_*k*_(*Genome*) and its traversal to perform *disembroiling transformations* (on both the graph and the genome) that substitute some pairs of similar regions in *Genome* by pairs of identical regions. Figure 3 illustrates a simplified case when GenomeDecoder, similarly to DRIMM-Synteny and Sibelia, uses bubbles in the de Bruijn graph to identify similar regions. This operation increases the number of shared *k*-mers in *Genome* and transforms it into a new disembroiled genome that is similar to *Genome* but has a more discernable block structure.

Figure 3 illustrates how GenomeDecoder collapses *simple bubbles* formed by pairs of parallel edges. In the case of HRRs, it is important to define *complex bubbles* and classify them into similar bubbles (that should be collapsed) and dissimilar bubbles (that should not be collapsed). GenomeDecoder extends the set of collapsible bubbles as compared to the graph simplification procedure in DRIMM-Synteny, Sibelia, and algorithm from Jiang *et al.* (2007). It also changes the way of extracting blocks from the de Bruijn graph (as compared to simply outputting the edge labels of the de Bruijn graph) to minimize the number of distinct letters in the ‘blocks alphabet’ and thus simplifying the downstream analysis. GenomeDecoder iteratively performs graph transformations by increasing the *k*-mer size at each iteration until it reaches a large value *K*, the user-specified lower bound for the minimum block size. For example, *K* is often set as 1–2 kb in SD analysis (Jiang *et al.* 2007, Pu *et al.* 2018) and as 0.5 Mb in genome rearrangement analysis (Pevzner and Tesler 2003, Bourque *et al.* 2004).

**Figure 4.**
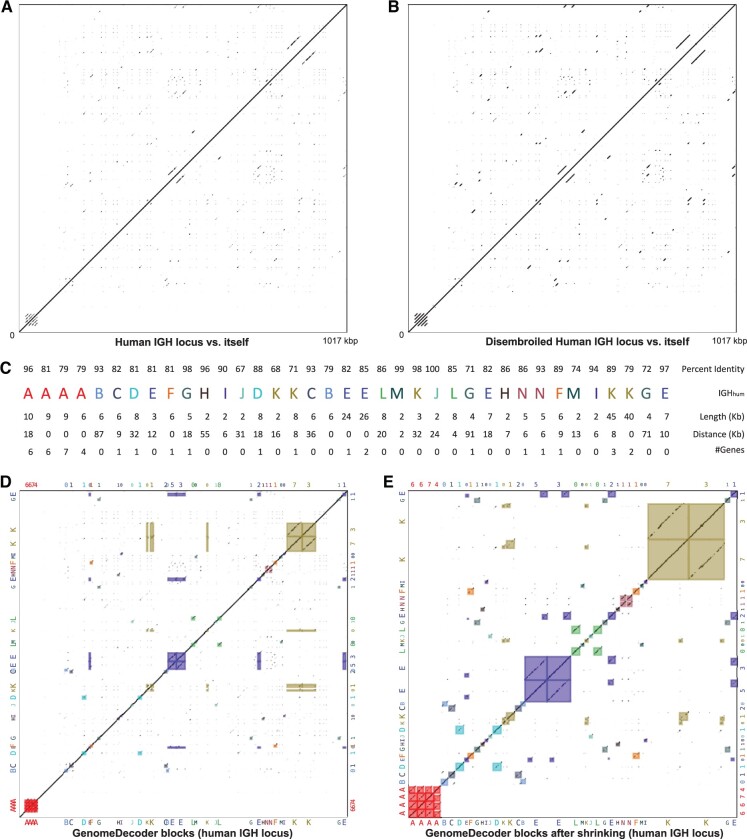
Dot-plot of IGH_hum_ against itself (A), dot-plot of the disembroiled IGH_hum_ against itself (B), the block decomposition of IGH_hum_ (C), the block-plot depicting each pair of block-instances in IGH_hum_ as a rectangle (D), and the scaled block-plot of IGH_hum_ (E). (A) The dot-plot of IGH_hum_ against itself. (B) The dot-plot of the disembroiled IGH_hum_ against itself. The imperfect repeats with mismatches and indels in IGH_hum_ are transformed into identical repeats in the disembroiled sequence. Although the two dot-plots look similar, the blocks in the disembroiled sequence are easier to infer. (C) Sequence IGH_hum_ in the block alphabet (reproduced from Fig. 1 for convenience). (D) Block-plot of IGH_hum_ against itself. The block-plot superimposes block-rectangles with the standard dot-plot for an improved visualization. Each block-rectangle depicts a pair of block-instances: an instance of a block in the decomposition on the bottom *x*-axis (with corresponding number of IGH genes on the top *x*-axis) and an instance of the same block in the decomposition on the left *y*-axis (with corresponding number of IGH genes on the right y-axis). The colors of the block-rectangles match their colors in (C). The boundaries of the block-rectangles are adjusted as described below so that both sides become similar in length. (E) Scaled block-plot of IGH_hum_ against itself is derived from the block-plot in (D) by removing all non-repetitive regions. It provides a better visualization since it shrinks the regions lying outside the repetitive blocks. All dot-plots are generated with the window size equal to 30 bp.

Although the original *Genome* and the disembroiled *Genome-DIS* are similar (Fig. 4A and B), the blocks in the disembroiled genome (in difference from the initial genome) represent perfect matches and thus are easily discernible using the graph DB_*K*_(*Genome-DIS*). GenomeDecoder also constructs the *block graph* DB*_K_(*Genome-DIS*) obtained by deleting all edges of multiplicity 1 from DB_*K*_(*Genome-DIS*). The block graph compactly represents all repeated blocks in *Genome-DIS*. After generating blocks in *Genome-DIS*, GenomeDecoder uses edlib (Sosic and Sikic 2017) to align the original genome against the disembroiled genome and thus ‘lift’ the blocks from the disembroiled genome to the original genome (alternatively, a user has an option of using UniAligner (Bzikadze and Pevzner 2023) for alignment).

The subsections below describe how GenomeDecoder transforms inexact genomic repeats into exact ones by using more complex graph transformations than the ones used in DRIMM-Synteny and Sibelia.

### 2.2 Collapsible bubbles

Given a path *P* in the DB graph, we denote the sequence spelled by this path as seq(*P*). Given an edge-weighted graph and its traversal, we say that two subpaths of this traversal form a *bubble* if they (i) start at the same vertex, (ii) end at the same vertex, (iii) share no other vertices. Two sequences are classified as *similar* if the percent identity between them exceeds a threshold *sim_strong_* (default value 90%) and as *weakly similar* if the percent identity between them exceeds a threshold *sim_weak_* (default value 65%).

We classify a bubble formed by two paths with *N* and *M* edges as an *N-M-bubble* and define its complexity as max{*N*, *M*}. A 1–1-bubble is referred to as a *simple bubble* and all other bubbles are referred to as *complex bubbles*. We classify a complex *N-M*-bubble as *short* if its complexity does not exceed a threshold *bubble_max_* (default value 4). We define the multiplicity of a path as the smallest multiplicity of its edges.

A simple bubble in a graph DB_*k*_(*Genome*) is classified as *collapsible* if its two edges *e*_1_ and *e*_2_ spell similar sequences. Given a collapsible simple bubble, we define a *bubble collapsing* operation that substitutes each substring of *Genome* spelled by *e*_1_ by the substring spelled by *e*_2_ (without loss of generality, we assume ties are resolved arbitrarily and that |seq(*e*_1_)| ≤ |seq(*e*_2_)). The bubble collapsing operation transforms a string-set *Genome* into another string-set *Genome’* and transforms the graph DB_*k*_(*Genome*) into the graph DB_*k*_(*Genome’*) by removing the ‘shorter’ edge of the bubble and adding its multiplicity to the multiplicity of its longer edge.

A complex bubble in a weighted graph is classified as *collapsible* if its two paths are weakly similar. Given a collapsible complex bubble in DB_*k*_(*Genome*) formed by paths *P*_1_ and *P*_2_, we define a *bubble collapsing* operation as substituting each substring of *Genome* spelled by *P*_1_ by the substring spelled by *P*_2_ (without loss of generality, we assume that |seq(*P*_1_)| ≤ |seq(*P*_2_)). In the vast majority of cases, this operation subtracts multiplicity(*P*_1_) from multiplicities of all edges in *P*_1_ and adds multiplicity(*P*_1_) to all edges in *P*_2_.

### 2.3 Disembroiled graphs

GenomeDecoder transforms inexact genomic repeats into exact ones by iteratively increasing the *k*-mer-size used for constructing the de Bruijn graph and disembroiling the resulting graphs. The pseudocode of the disembroiling algorithm is given in Supplementary Note S1.

### 2.4 Generating blocks in the disembroiled genome and representing a genome in the block alphabet

The disembroiled graph DB_*K*_(*Genome-DIS*) simplifies inference of blocks since each edge in this graph gives rise to a block. However, the resulting block-set includes overlapping blocks since edges that start/end at the same vertex result in blocks that overlap by *K* nucleotides. Even though one can ignore such overlaps when the *K*-mer size is much smaller than the block size, ignoring overlaps between blocks leads to complications in analyzing HRRs. Our goal is to partition the disembroiled genome into non-overlapping blocks.

Supplementary Note S2 describes the block generation algorithm in GenomeDecoder. Supplementary Note S3 describes how GenomeDecoder represents a genome in the block alphabet. In contrast to approaches in DRIMM-Synteny, Sibelia, SDquest, BISER, and the algorithm by Jiang *et al.* (2007) (that generate blocks in such a way that all instances of the same block have similar lengths), different instances of the same block generated by GenomeDecoder may have widely different lengths. This feature is important since it reduces the number of blocks in the block decomposition. We note that this representation maintains all information about the genomic architecture since it provides starting and ending positions of the partial instances of each block.

### 2.5 Visualizing the block decomposition as a block-plot

The *block-plot* visualizes the constructed blocks by superimposing the standard dot-plot with *block-rectangles* that represent each pair of instances of the same block (Fig. 4D). For every two instances of the same block starting at positions *i*_1_ and *i*_2_ in the genome (of length *l*_1_ and *l*_2_, respectively), we define their block-rectangle in the dot-plot as the rectangle with its bottom left corner located at the coordinate (*i*_1_, *i*_2_) and its upper right corner located at the coordinate (*i*_1_+*l*_1_, *i*_2_+*l*_2_). The color of the block-rectangle is defined as the assigned color of the corresponding block in the block decomposition. It turned out that the blocks generated by GenomeDecoder capture nearly all sufficiently long similar regions (hardly any similar regions are located outside block-rectangles) and transform them into exact repeats in the disembroiled graph (diagonals within block-rectangles).

### 2.6 Visualizing block decompositions using scaled block-plots

To improve visualization of duplicated regions, we generate the *scaled block-plot* obtained from the block-plot by removing non-repetitive regions and modifying the block boundaries (Fig. 4E). Since block-instances of the same block may have vastly different lengths, the similarity between some block-instances may be more adequately represented by fitting (or even overlapping) alignment rather than their global alignment. Supplementary Note S4 describes how we transform each block-rectangle into a *block-square* to capture only the aligned regions between the block-instances. The scaled block-plot is formed by substituting each block-rectangle by its block-square.

### 2.7 Generating block decomposition for multiple genomes

Generating block decomposition for multiple genomes. Supplementary Note S5 describes how GenomeDecoder constructs the blocks for multiple genomes using the same ‘disembroiled genome’ approach but applies it to the combined string-set of all these genomes.

## 3 Results

### 3.1 Datasets

We analyzed the following DNA sequences:

#### 3.1.1 Primate heavy chain immunoglobulin loci

Heavy chain immunoglobulin (IGH) loci in mammalian genomes represent complex HRRs with poorly understood history of duplications. We analyzed haplotypes of human, Bornean orangutan, Sumatran orangutan, bonobo, and gorilla IGH loci assembled by the Primate T2T consortium (Yoo *et al.* 2024):

- IGH_hum_—human IGH locus of length 1017 kb from the reference human genome hg38
- IGH_humT2T_—human IGH locus of length 1161 kb assembled by the T2T consortium (Nurk *et al.* 2022)
- IGH_B.orang_—Bornean orangutan IGH locus of length 1416 kb (haplotype 1)
- IGH_S.orang_—Sumatran orangutan IGH locus of length 1342 kb (haplotype 1)
- IGH_bonobo_—bonobo IGH locus of length 1183 kb (haplotype 2)
- IGH_gorilla_—gorilla IGH locus of length 977 kb (haplotype 1)

The non-human primate IGH loci were assembled by the Primate T2T consortium. IGH (V, D, and J) genes in IGH_hum_ and IGH_humT2T_ were inferred based on the NCBI genes annotation for GRCh38 and T2T-CHM13v2.0 human genomes, respectively (Supplementary File S1). All IGH genes in ape genomes were predicted by the Primate T2T consortium using IGDetective (Sirupurapu *et al.* 2022) and Digger (Lees *et al.* 2024) tools.

#### 3.1.2 Primate MHC loci

The major histocompatibility complex (MHC) locus is a highly repetitive region that encodes cell surface proteins essential for the vertebrate immune system. We analyzed the following primate MHC loci:

- MHC_humT2T_h—human MHC locus of length 3936 kb assembled by the T2T consortium (Nurk *et al.* 2022).
- MHC_B.orang_—Bornean orangutan MHC locus of length 4400 kb (haplotype 1) assembled by the Primate T2T consortium (Yoo *et al.* 2024).

#### 3.1.3 Human chromosomes

We analyzed chromosome 20 and X from two human genomes.

- HUM_20_—chromosome 20 of lengths 64.4 Mbp from the reference human genome hg38
- HUMT2T_20_—chromosome 20 of lengths 66.2 Mbp assembled by the T2T consortium (cell line CHM13)
- HUM_X_—chromosome X of lengths 156.0 Mbp from the reference human genome hg38
- HUMT2T_X_—chromosome 20 of lengths 154.3 Mbp assembled by the T2T consortium (cell line CHM13)

Below we analyze primate IGH loci and human chromosomes. Supplementary Note S6 analyzes primate MHC loci.

### 3.2 Comparing block generation tools

Benchmarking block generation tools is a challenging task since they use different parameters and optimize different implicitly defined (or undefined) objective functions (Liu *et al.* 2018). We illustrate this challenge by benchmarking halSynteny, SDquest, and Sibelia. We did not benchmark other SD detection tools, e.g. MashMap2 (Jain *et al.* 2018) and ASGART (Delehelle *et al.* 2018) because they focus on accurate SD detection rather than decomposing a genome into blocks and, according to Iseric *et al.* (2022), underperform compared to SDquest (Pu *et al.* 2018) and SEDEF (Numanagić *et al.* 2018) that are able to detect ancient SDs. Since the large fraction of GenomeDecoder’s runtime is spent generating alignments, Supplementary Note S7 describes benchmarking various alignment tools in comparing long strings.

**Figure 5.**
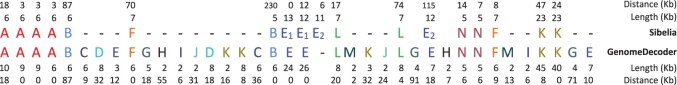
Comparison of blocks generated by GenomeDecoder and Sibelia for the IGH_hum_ locus. Dashes (‘-’) represent gaps in the alignment of block-instances.

Supplementary Note S8 illustrates that halSynteny fails to generate non-overlapping blocks. We compared Sibelia and GenomeDecoder by analyzing their block decomposition of IGH_hum._  Figure 5 compares 7 blocks generated by Sibelia (A, B, F, E, K, L, and N) with 14 blocks generated by GenomeDecoder that are shown in Fig. 1. Supplementary Note S9 compares these two block decompositions and illustrates some deficiencies in block decompositions generated by Sibelia.

SDquest and BISER generate excellent yet over-fragmented block decompositions (Supplementary Note S10). We note that GenomeDecoder has a slightly different focus than SDquest/BISER as it aims to generate compact rather than over-fragmented block decompositions (even at the expense of coarsening block decompositions generated by SDquest/BISER) to simplify the downstream analysis of the genomic architectures.

Supplementary Note S10 illustrates that SDquest decomposed IGH_hum_ into 567 block-instances of 104 blocks, including 78 block-instances of 32 large blocks that are longer than 2 kb (compared with only 37 block-instances of 14 blocks by GenomeDecoder). This over-fragmentation may be explained by the fact that SDquest, in difference from GenomeDecoder and Sibelia, uses a single *k*-mer size instead of iteratively increasing the *k*-mer size. In a sense, SDquest blocks decompositions may be similar to the GenomeDecoder/Sibelia block decompositions generated for a single *k*-mers size with an additional benefit of capturing ancient highly diverged blocks that GenomeDecoder and Sibelia may miss (Pu *et al.* 2018).

The SDquest developers noticed this over-fragmentation and described an approach for enlarging their blocks by combining them into larger *SD-units*. Although this procedure decomposed IGH_hum_ into a smaller number of SD-units (60), it did not completely address over-fragmentation. Since SDquest generates accurate yet over-fragmented block decompositions, we have modified GenomeDecoder for enlarging blocks generated by SDquest, implicitly adding the benefit of iterative graph simplifications to SDquest (Supplementary Note S10).

### 3.3 Block decomposition reveals rapid birth/death of IGH genes

**Figure 6.**
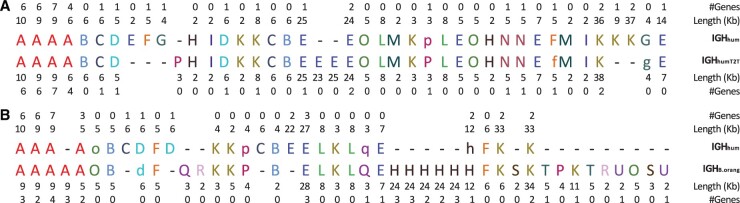
Differential analysis of blocks in IGH_hum_ vs. IGH_humT2T_ (A) and IGH_hum_ vs. IGH_B.orang_ (B). (A) 15 blocks form 39 and 37 block-instances for IGH_hum_ and IGH_humT2T_, respectively. Block decomposition illustrates large variations on the copy numbers and gene numbers between IGH_hum_ and IGH_humT2T_. For example, F/G/K blocks have 1/1/2 additional copies and 1/1/3 additional V genes in IGH_hum_ as compared to IGH_humT2T_. The figure provides information about the length of each block-instance, and the number of IG genes in each block-instance. The block alphabet derived for the single IGH_hum_ sequence (Fig. 1) differs from the block alphabet derived for the same sequence in the case when we compare it with a different sequence IGH_humT2T_ because we apply the same default parameters to different graphs DB_*k*_(IGH_hum_) and DB_*k*_(IGH_hum_+IGH_humT2T_). For example, a short block J in Fig. 1 ‘disappeared’ in (A) because its length (2 kb) is close to the threshold *k*-mer size 2 kb. (B) Human and orangutan IGH loci in the alphabet of 16 blocks that form 26 and 40 block-instances in the induced alignment of IGH_hum_ and IGH_B.orang_, respectively (only 22 of these block-instances are aligned against each other).

Figure 6A illustrates large variations in the architectures of two human IGH loci (IGH_hum_ and IGH_humT2T_) and reveals that multiple SDs/TDs led to a rapid birth/death of IG genes within the human population. For example, F, G, and K blocks have 1 + 1 + 2 additional block-instances in IGH_hum_ and 1 + 1 + 3 additional V genes as compared to IGH_humT2T_.

Figure 6B shows the human and orangutan IGH loci in the block alphabet and reveals many non-aligned blocks. For each edge in the graph DB_*k*_(*Genome*_1_+*Genome*_2_), we represent its multiplicity as (*m*_1_.*m*_2_), where *m*_1_ and *m*_2_ are its multiplicities in *Genome*_1_ and *Genome*_2_, respectively. An edge with multiplicity (*m*_1_.*m*_2_) in the graph DB_*K*_(*Genome*_1_-*DIS*+*Genome*_2_-*DIS*) is called *imbalanced* if *m*_1_≠*m*_2_. A block is classified as imbalanced if one of its edges is imbalanced. Imbalanced blocks reveal differences in SDs across two genomes.

Figure 6B demonstrates that, even for close primate species, most blocks in the IGH locus are imbalanced, revealing a very high rate of SDs/TDs. We emphasize that even though the alignment of human and orangutan IGH loci in the block alphabet (Fig. 6B) becomes so obvious that it can be constructed by hand, adequate alignment of HRRs in the nucleotide alphabet is a non-trivial task (Bzikadze and Pevzner 2023, Liao *et al.* 2023).

### 3.4 Block decomposition reveals large structural variations in human chromosomes and prevalence of reverse tandem duplications

**Figure 7.**
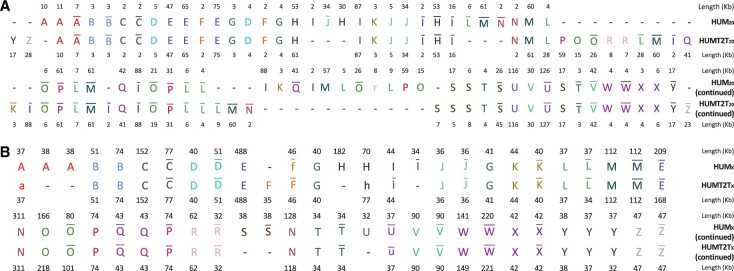
Differential analysis of blocks in human chromosomes HUM_20_ and HUMT2T_20_ (A) and HUM_X_ and HUMT2T_X_ (B). Figure provides information about the length of each block-instance. Reverse complementary block-instances are shown using an overline on the corresponding block letters. All blocks longer than 2 kb are shown in (A) but only blocks longer than 32 kb are shown in (B).

Figure 7A illustrates large variations in genome architectures of chromosomes HUM_20_ and HUMT2T_20_ that are represented in the alphabet of 26 blocks. These blocks form 69 (70) block-instances in the HUM_20_ (HUMT2T_20_) that vary in length from 2 to 127 kb. However, only 51 out of these block-instances are aligned against each other.

Human chromosome 20 has a relatively simple architecture as compared to other human chromosomes. Figure 7B presents the block decomposition of chromosomes HUM_X_ and HUMT2T_X_ generated by GenomeDecoder. It only shows large blocks (longer than 32 kb) since the block decomposition for shorter blocks is so complex that its visualization becomes difficult. GenomeDecoder generated 26 large blocks forming 53 and 47 block-instances in HUM_X_ and HUMT2T_X_, respectively. Figure 7B illustrates that *reverse tandem duplications* (such as the block C followed by its reverse-complementary sequence with some mutations) are common elements of the human chromosomal architecture. Even when the analysis is limited to large blocks, HUM_X_ and HUMT2T_X_ differ from each other in the number of instances of blocks A, F, I, S, and U. Supplementary File S2 presents decomposition of HUM_X_ and HUMT2T_X_ into shorter blocks (at least 2 kb) and illustrates that these chromosomes have very different architectures (139 blocks that form 413 and 371 block-instances for HUM_X_ and HUMT2T_X_, respectively).

### 3.5 Block decomposition reveals selective pressure in the IGH loci

Here, we illustrate how block decomposition contributes to gene prediction in IG loci. We limit analysis to D genes that are notoriously difficult to predict by the existing IG gene prediction tools (Safonova and Pevzner 2020, Sirupurapu *et al.* 2022, Lees *et al.* 2024). D genes are located in the IGHD locus and 23 out of 27 human D genes in IGH_hum_ occur within the four-unit tandem repeat AAAA within this locus (Fig. 1). We denote its four units as A1_hum_/A2_hum_/A3_hum_/A4_hum_. We also analyzed this tandem repeat in Bornean orangutan (units A1_B.orang_–A5_B.orang_), Sumatran orangutan (units A1_S.orang_–A5_S.orang_), gorilla (units A1_gorilla_–A5_gorilla_), and bonobo (units A1_bonobo_–A6_bonobo_). Each of these 4 + 5 + 5 + 5 + 6 = 25 *A-blocks* was aligned against A1_hum_ using the edlib tool (Sosic and Sikic 2017).

**Figure 8.**
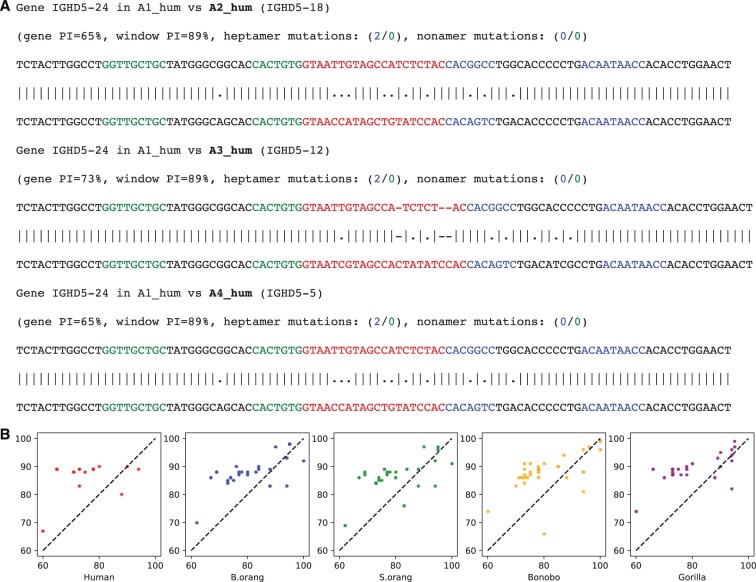
Alignments of the block A1_hum_ against blocks A2_hum_, A3_hum_, and A4_hum_ centered at gene IGHD5-24 (A) and analysis of selective pressure in primate IGHD loci (B). (A) The D genes within a 100 bp long window are centered in each sequence. The downstream (upstream) heptamers and nonamers forming the Recombination Signal Sequence (RSS) are highlighted in different colors. In the example shown, the alignment between A1_hum_ and A2_hum_/A3_hum_/A4_hum_ aligns the gene IGHD5-24 with D genes IGHD5-18/IGHD5-12/IGHD5-5. (B) The block-based alignments reveal the selective pressure on D genes in primate IGH loci. Each block-annotated D gene is represented as a point (*x*, *y*) in 2D, where *x* represents the gene PI between this gene and the corresponding D gene from A1_hum_ and *y* represents the window PI between the 100 bp long window centered at this gene and the corresponding window from A1_hum_.

There are six annotated D genes in the block A1_hum_ (IGHD5-24, IGHD4-23, IGHD3-22, IGHD2-21, IGHD1-20, IGHD6-19). Twenty-four pairwise alignments of this block against various A-blocks allow us to trace the birth/death of D genes. Figure 8A shows alignments of IGHD5-24 in A1_hum_ against A2_hum_, A3_hum_, and A4_hum_ within a 100 bp window centered at IGHD5-24. The *gene PI* in this figure refers to the percent identity of the alignment between IGHD5-24 in A1_hum_ and the gene it aligns to. The *window PI* refers to the percent identity of the alignment between the 100 bp long window centered at this gene in A1_hum_ and the window it aligns to. The case when the gene PI is significantly larger than the window PI suggests selective pressure on this gene (Kondrashov 2012).

Figure 8A demonstrates that evolution of the IGHD5-24 gene was subjected to a strong selective pressure that resulted in an increased mutation rate in this gene as compared to the mutation rate in the surrounding window. Indeed, the average percent identity between IGHD5-24 in A1_hum_ and its aligned regions in A2_hum_, A3_hum_, and A4_hum_ (68%) is much smaller than the average percent identity between the surrounding regions (89%). The same conclusion holds for 5 out 6 D genes in the block A1_hum_: IGHD4-23 (75% vs 87%), IGHD3-22 (71% vs 82%), IGHD2-21 (76% vs 89%), and IGHD6-19 (81% vs 89%). In contrast, IGHD1-20 turned out to be very conserved when aligned to A2_hum_ (only 2 substitutions) and A3_hum_ (only 1 substitution). Further analysis revealed that this D gene is exceptionally conserved in all considered primate IGH loci and it is the only D gene that is more conserved than the surrounding window. Figure 8B confirms this conclusion by illustrating a high number of genes that have gene PI lower than window PI.

### 3.6 Block decomposition contributes to gene identification in IGH loci

Alignments of some gene-centered windows reveal that an annotated IG gene in A1_hum_ was disrupted in some A-blocks. Figure S5 in Supplementary Note S7 shows alignments of genes IGHD3-22 and IGHD1-20 in A1_hum_ against A4_hum_. The edlib alignment of IGHD3-22 has a high window PI of 89% and reveals a related D gene in A4_hum_ that differs from IGHD3-22 by 2 indels and 5 mismatches. In contrast, the edlib alignment of IGHD1-20 has a very low window PI of 36%, suggesting that IGHD1-20 was disrupted in A4_hum_.

Recently, the Primate T2T consortium annotated genes in all primate species using IGDetective (Sirupurapu *et al.* 2022) and Digger (Lees *et al.* 2024). This annotation includes 24, 23, 34, and 25 D genes in Bornean orangutan, Sumatran orangutan, bonobo, and gorilla IGHD loci, respectively, out of which 20, 19, 29, and 19 lie within the A-blocks of respective genomes. The number of annotated D genes within a single A-block varies from 2 to 7, illustrating the birth/death of D genes.

We say that a D gene in A1_hum_ is *disrupted* in a given A-block if the window PI of its alignment against this block falls below a threshold *minPI* (default value 60%) or if its RSS differs from the RSS of the corresponding gene in A1_hum_ by more than *maxDiff* substitutions in heptamers and nonamers (default value *maxDiff* = 2). Otherwise, we classify a D gene as *block-annotated*.

**Figure 9.**
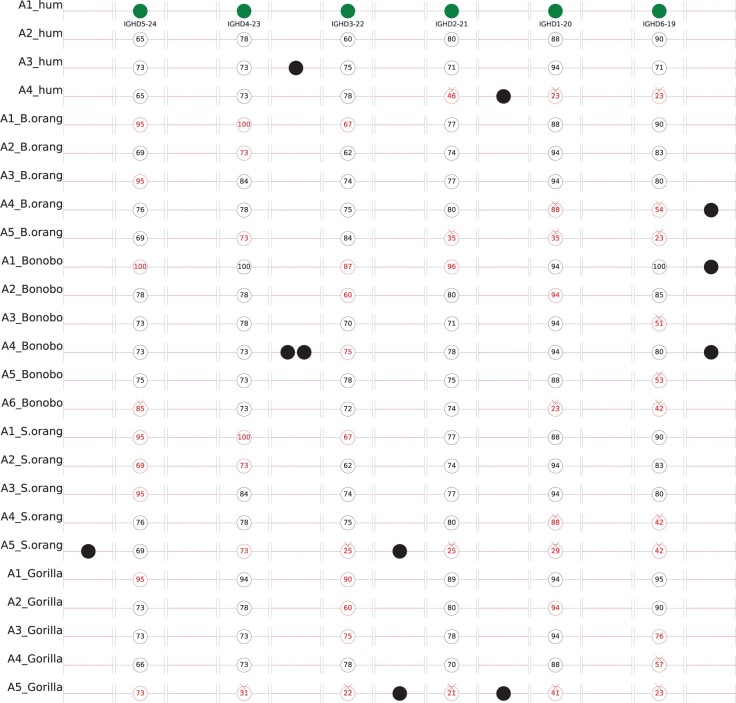
Schematic visualization of the alignment of A1_hum_ against all A-blocks in primate species along with information about D-genes located within these A-blocks. Each of 24 A-blocks in the primate IGH loci was aligned against A1_hum_. An alignment of the entire A1_hum_ block against each primate A-block aligns each of six annotated D genes in A1_hum_ against a short segment in this block that often reveals a putative primate D gene. Crosses schematically show disrupted genes within A-blocks. Hollow circles that are not highlighted with color (such as all circles in A2_hum), and all solid filled circles (such as the first circle in A1_hum and the third circle in A3_hum) show all the 23 + 20 + 19 + 29 + 19 annotated D genes within all A-blocks in human, Bornean orangutan, Sumatran orangutan, bonobo, and gorilla IGH loci, respectively. Hollow circles that are not highlighted with color show the annotated genes that were also predicted by our block-based alignment against A1_hum_ while solid filled circles in lines 3, 4, 8, 10, 13, 20, and 25 (such as the third circle in A3_hum) show the annotated genes that the block-based approach failed to match. The hollow circles highlighted with color (such as the fourth circle in A4_hum, and the first circle in A1_B.orang) show the D genes predicted by our block-based approach but missed in the existing annotations. The numbers within the hollow circles show the percent identity with the aligned D gene in A1_hum_.

Out of 6*24 = 144 alignment windows of the annotated D genes in A1_hum_, 25 were disrupted (crosses in Fig. 9), 93 were predicted by the Primate T2T consortium/NCBI (hollow circles that are not highlighted with color in Fig. 9), and 26 were block-annotated but missed by Primate T2T consortium/NCBI annotations (hollow circles that are highlighted with color and not marked by crosses in Fig. 9). Solid circles that are not highlighted with color in Fig. 9 show 11 D genes annotated by the Primate T2T consortium/NCBI that are not orthologous/paralogous to the annotated D genes in A1_hum_ and thus cannot be ‘lifted’ from the constructed block alignments.

Figure 9 illustrates that block-annotated genes reveal many D genes missed by the state-of-the-art tools for predicting IG genes. It is not surprising since these tools have to be very conservative to maintain the trade-off between false positive and false negative gene predictions. However, even with this conservative trade-off, some D genes, represented by solid black circles in Fig. 9, may be false positives.

### 3.7 Block-based analysis suggests that immunoglobulin loci are subjected to diversifying selection

All analyzed primate IGH loci feature a significantly elevated percentage of SDs/TDs (≈35%) compared to the average percentage of duplications in the primate genomes (≈6%). Moreover, our analysis of the primate IGH loci revealed considerable variations in block decompositions across different primates and even within each individual primate population.

While previous studies did not provide block decompositions of the human IGH loci, they identified large SVs in these loci using existing SV identification tools (Ebert *et al.* 2021, Rodriguez *et al.* 2023). These studies, along with our own, raise the question of the evolutionary benefits provided by maintaining population-wide structural diversity in IG loci as opposed to the highly conserved architectures of other loci. Since this question remained unanswered, we hypothesize that high variability of human IG loci provides a population-wide selective advantage in mounting antibody response to location-specific and new infections. Supplementary Note S11 ‘Diversifying selection in immunoglobulin loci’ describes this hypothesis.

## 4 Discussion

SDs and TDs play crucial roles in genome evolution. While numerous studies have focused on identifying and mapping blocks of SDs and TDs to decompose genomes, current algorithms struggle with highly complex genomic regions like IG loci. This is unfortunate since studies of SD/TD evolution face multiple challenges, e.g. even identification of orthologous/paralogous genes in SDs/TDs remains an open problem.

The challenge in developing block decomposition algorithms for highly repetitive regions also stems from the lack of a universally accepted objective function for assessing the quality of block decompositions. Without a clear objective function, comparing different algorithms becomes difficult, making downstream biological analysis a key factor in evaluating each novel block decomposition tool. In this context, we presented both GenomeDecoder and downstream analysis of genomic architectures it generated. Specifically, we showed that it revealed rapid birth/death of IG genes, prevalence of reverse TDs, evidence of diversifying selection in IG loci, previously undetected IG genes, etc These findings highlight potential of GenomeDecoder in advancing our understanding of complex genomic regions.

Although GenomeDecoder is based on the same key ideas that were implemented in DRIMM-Synteny (DBG simplification) and Sibelia (iterative DBG simplification), our benchmarking suggests that it generates more adequate representations of genome architectures. After the genomes are decomposed into blocks, alignment of sequences in the block alphabet becomes much simpler than their alignment in the nucleotide alphabet, thus addressing the difficult problem of finding orthologous/paralogous genes in HRRs. We illustrated how block-based alignments lead to predictions of novel primate IG genes (that were missed by existing IG gene prediction tools) and revealed selective pressure on IG genes. Another contribution of GenomeDecoder is a new approach for generating non-overlapping blocks from the de Bruijn graph and minimizing the number of blocks for a compact representation of a genome in the block alphabet. This compact representation is important since over-fragmented block decompositions complicate the downstream analysis.

Analysis of genome rearrangements often becomes unreliable in the case of incomplete and error-prone assemblies (Alekseyev and Pevzner 2009). As a result, although many mammalian genomes have been assembled in the first two decades after completing the mouse genome project (Waterston *et al.* 2002), the inference of the rearrangement history of mammals remains an open problem. Recent ‘complete genomics’ projects have addressed this limitation and opened a possibility to generate accurate rearrangement-based evolutionary scenarios using complete genomes (Rhie *et al.* 2021). However, such rearrangement analysis is only possible after the inference of blocks.

One limitation of genome rearrangement tools is that they are mainly limited to analyzing non-duplicated blocks appearing only once in each genome (Bourque and Pevzner 2002, Alekseyev and Pevzner 2009). Since including duplicated blocks in this analysis faces significant challenges (Avdeyev *et al.* 2016), evolutionary studies of HRRs, such as immunoglobulin loci, remained difficult. First, it was unclear how to represent such regions in the alphabet of blocks, and second, analyzing evolution of such regions, even after representing them in the alphabet of blocks, remains an open algorithmic problem. GenomeDecoder addresses the first problem and thus opens a possibility of exploring various approaches to the second problem.

## Supplementary Material

btaf058_Supplementary_Data

## Data Availability

All IGH, MHC, and human chromosomes have been uploaded to https://doi.org/10.5281/zenodo.14753844. GenomeDecoder is available at https://github.com/ZhangZhenmiao/GenomeDecoder. The block-plot visualization code is available at https://github.com/IshaanSD/BlockPlot.
