# Supplementary material for: GenomeDecoder: inferring segmental duplications in highly repetitive genomic regions
Source: Bioinformatics. 2025 Feb 5;41(2):btaf058. doi: 10.1093/bioinformatics/btaf058 (PMC11842051; doi:10.1093/bioinformatics/btaf058)
Supplement: btaf058_Supplementary_Data [file btaf058_supplementary_data.zip › Supplementary_Notes.pdf]

Supplementary Notes

- S1: Constructing disembroiled graphs
- S2: Generating blocks in the disembroiled genome
- S3: Representing a genome in the block alphabet
- S4: Transforming block-rectangles into block-squares
- S5: GenomeDecoder algorithm for multiple genomes
- S6: Comparing genomic architectures of the primate MHC loci
- S7: Benchmarking chromosome-scale alignment tools
- S8: Benchmarking halSynteny
- S9: Benchmarking Sibelia
- S10: Benchmarking SDquest
- S11: Why are there so many SVs in the primate immunoglobulin loci?
- S12: Parameters of GenomeDecoder
- References

Supplementary Note S1: Constructing disembroiled graphs

Why is it important to construct the disembroiled graphs in an iterative fashion? We illustrate the benefits of the iterative block decomposition using the IGH_{hum} locus. When the *k*-mer size is small ($k = 2*\log_4 | IGH_{hum} |$), the graph $DB_k(IGH_{hum})$ has 10828 edges and 7048 vertices while the disembroiled graph $DB_k(IGH_{hum}\text{-DIS})$ has 8710 edges and 5658 vertices). Thus, the disembroiled graph for a single (small) *k*-mer size does not yield compact block decomposition. When the *k*-mer size is large (*K*=2000), the graph $DB_K(IGH_{hum})$ has only 2 edges and 4 vertices while the graph $DB_K(IGH_{hum}\text{-DIS})$ has 128 edges and 88 vertices. Thus, while the disembroiled graph for a single (large) *k*-mer size does not reveal block decomposition, the disembroiled graph $DB_K(IGH_{hum}\text{-DIS})$ constructed in the iterative fashion does, motivating the iterative approach for constructing block decompositions.

Given an integer *K*, we define the *duplication ratio* of a string-set *Genome* (referred to as $DupRatio_k(Genome)$) as the fraction of non-unique *k*-mers in *Genome*. The duplication ratio of the disembroiled genome $DupRatio_{2000}DupRatio(IGH_{hum}\text{-DIS})$=0.26, while the duplication ratio of the original genome $DupRatio_{2000}(IGH_{hum})$=0. Figure 2 illustrates that imperfect repeats in IGH_{hum} have been transformed into perfect repeats in the disembroiled genome $IGH_{hum}\text{-DIS}$, resulting in a relatively simple graph $DB_K(IGH_{hum}\text{-DIS})$. At a small *k*=31, $DupRatio_{31}(IGH_{hum}\text{-DIS})$=0.36, close to the fraction of the total length of all duplicated blocks over the total length of IGH_{hum}.

The Disembroil algorithm. The **Disembroil** algorithm (pseudocode below) transforms inexact genomic repeats into exact ones by iteratively increasing the *k*-mer-size used for constructing the de Bruijn graph. Initially, it sets a small *k*-mer size (default value $2*\log_4 |Genome|$) and doubles it in an iterative fashion (|*Genome*| stands for the total length of strings in the string-set *Genome*). Since low-complexity regions and common repeats (such as Alu repeats in the human genome) have many shared *k*-mers that confuse block generation, they are typically ignored by the block generation algorithms. GenomeDecoder achieves the same goal by identifying common repeats using RepeatMasker v4.1.5 (http://www.repeatmasker.org) and substituting them with random sequences.

The **Disembroil** algorithm takes a repeat-masked string-set *Genome*, integer *K* (minimal threshold on the length of the block with the default value 2 kb), and integer $bubble_{max}$ (upper threshold on the bubble complexity). It also uses external parameters sim_{strong} and sim_{weak} that define the concepts of similar and weakly-similar strings.

Given a bubble *B* in the de Bruijn graph *DBG* of a genome *Genome*, we denote the result of the bubble collapsing operation on *DBG* and *Genome* as **CollapseBubble**(*DBG*, *B*) and **DisembroilBubble**(*Genome*, *DBG*, *B*), respectively.

Disembroil(*Genome, K,* $bubble_{max}$)

```
    k ← 2*log4 |Genome|
  DBG ← DBk(Genome)
  while k < K
    while there are simple collapsible bubbles in DBG
      B ← an arbitrary simple collapsible bubble in DBG
      DBG ← CollapseBubble(DBG,B)
      Genome ← DisembroilBubble(Genome,DBG,B)
    k ← 2*k
  k ← 2*log4|Genome|
  while k < K
    while there are short collapsible bubbles in DBGraph
      B ← a short collapsible bubble of minimum complexity in DBG
      DBG ← CollapseBubble(DBG,B)
      Genome ← DisembroilBubble(Genome,DBG,B)
    k ← 2*k
return Genome
```

The Disembroil algorithm returns the disembroiled version of *Genome* that we denote as *Genome-DIS*. It also constructs graph DB_K(*Genome-DIS*) and the *block graph* DB^*_K(*Genome-DIS*) obtained by deleting all edges of multiplicity 1 from DB_K(*Genome-DIS*). The block graph compactly represents all repeated blocks in *Genome-DIS.*

Supplementary Note S2: Generating blocks in the disembroiled genome

Given a string-set *Genome,* GenomeDecoder constructs the disembroiled genome *Genome-DIS* and the block graph DB^*_K(*Genome-DIS*). It further partitions edges of the block graph into paths/cycles and classifies these paths/cycles as blocks in *Genome-DIS.*

Coloring edges and vertices of a graph forms a *partition* if (i) edges of each color form a simple path or a simple cycle, and (ii) internal vertices of each such path are colored by the same color, and (iii) all vertices of each such cycle, except for possibly a single vertex, are colored by the same color.

Edges of each colored path/cycle in this partition spell a block in *Genome* but the resulting blocks may overlap since they may share vertices. To generate non-overlapping blocks, GenomeDecoder shortens some of them as follows. If a colored path starts (ends) at a vertex of a different color, the linear block is shortened by removing its *K* initial (terminal) nucleotides. Similarly, if a colored cycle contains a vertex of a different color, the cyclic block is transformed into a linear one by deleting the *K* nucleotides that label this vertex. This approach ensures that the generated blocks do not overlap. The multiplicity of a block is defined as the maximum multiplicity of its edges.

We extend the generated set of repetitive blocks by non-repetitive blocks of multiplicity 1 in the graph DB_K(*Genome*) that were deleted from the block graph DB^*_K(*Genome*). If an edge corresponding to a non-repetitive block starts (ends) in a colored vertex, it is shortened by removing its *K* initial (terminal) nucleotides.

There are many ways to generate a partition of the block graph, e.g., by coloring edge by an individual color. To generate long blocks (and thus simplify the downstream analysis of the block decomposition), GenomeDecoder generates an *iterative partition* by iteratively removing a non-branching path/circle with a minimum multiplicity (the multiplicity is defined as the largest multiplicity of all the edges in the path/circle) from the graph and assigning it a specific color, until all edges have been assigned a color. Figure S1 illustrates seven blocks generated by GenomeDecoder, including a “red” block formed by five edges. This representation differs from the less compact “single-edge-single-block” representations generated by DRIMM-Synteny, Sibelia, and SDquest.

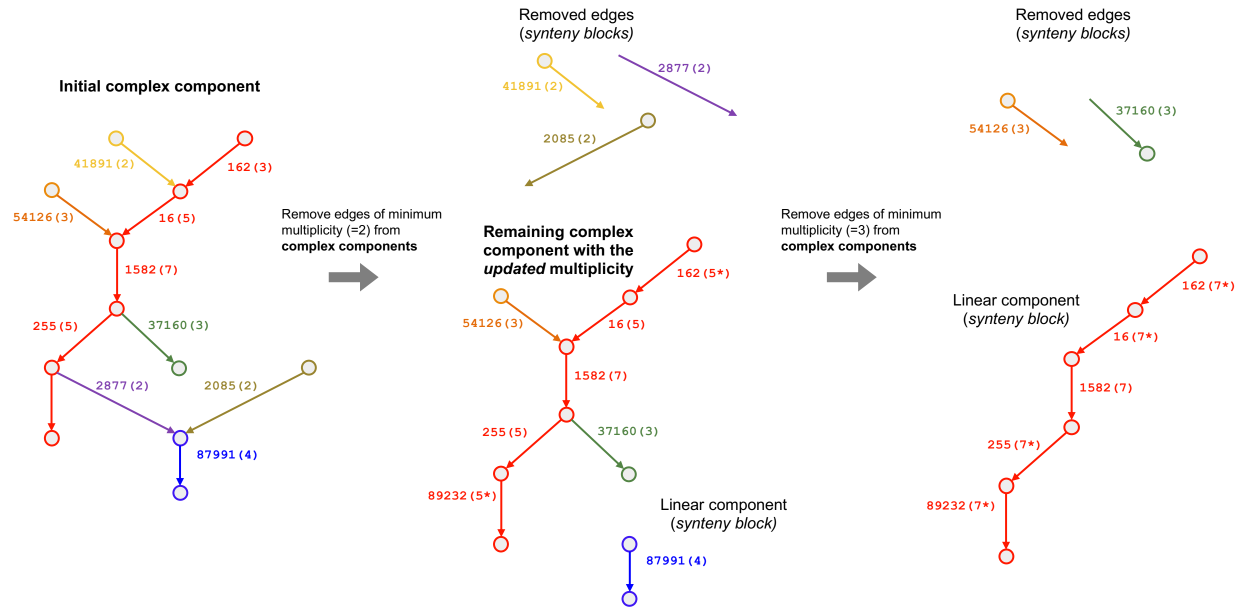

Figure S1. A connected component of the block graph of the human chromosome 20 in the human hg38 assembly and its decomposition into blocks. Each block corresponds to a path formed by edges of the same color. The label of each edge shows its length and multiplicity (in parenthesis). A star (*) after multiplicity means the multiplicity of this edge has been updated using the maximum multiplicity of the non-branching path it belongs to.

Supplementary Note S3: Representing a genome in the block alphabet

The string-set *Genome-DIS* defines a traversal of the graph DB_K(*Genome-DIS*) that can be used to "rewrite" this string-set in the alphabet of edges $e_1, \ldots e_n$. Note that some edges in this traversal (that belong to the graph DB^*_K(*Genome-DIS*)) are colored.

We further transform this traversal in the edge-alphabet into the traversal in the block-alphabet. We classify each contiguous run of edges of the same color (block) as an instance of this block and substitute the entire run with a single symbol representing this block. We note that such runs may represent partial rather than complete instances of a block. Thus, in contrast to block generation approaches in DRIMM-Synteny, Sibelia, and SDquest (that generate blocks in such a way that all instances of the same block have similar lengths), different instances of the same block generated by GenomeDecoder may have widely different lengths. For example, the fourth instance of the block A in Figure 1 is shorter than three previous instances (6 kb as compared to 10, 9, and 9 kb) since it represents a partial rather than complete instance of this block.

In practice, this approach significantly reduces the number of symbols in the block-alphabet, e.g., instead of representing a "genome as $\mathbf{ABC}_X\mathbf{BC}_Y\mathbf{CB}_Z\mathbf{B}$, GenomeDecoder defines a block **D=ABC** and represents this genome as $\mathbf{D}_X\mathbf{D}_Y\mathbf{D}_Z\mathbf{D}$ (X, Y, and Z stand for non-repetitive segments). This representation maintains all information about the genomic architecture since GenomeDecoder provides starting and ending positions of the partial instances of the block **D**.

Generating blocks in the original genome. GenomeDecoder uses the constructed blocks in the disembroiled genome to infer blocks in the original genome by "lifting" them from the disembroiled genome. It aligns the disembroiled genome *Genome-DIS* against the original *Genome* using edlib (Sosic and Sikic, 2017) and uses this alignment to define blocks in *Genome*. Each block-instance (a substring) in *Genome-DIS* is aligned against a substring in *Genome* that represents the same block-instance. Pseudocode below describes how GenomeDecoder decomposes a string-set *Genome* into blocks.

```
GenomeDecoder(Genome, K, bubble_max)
  Genome-DIS <- Disembroil(Genome, K, bubble_max)
  construct a greedy partition of the graph DB*_K(Genome-DIS)
```

```
transform each uni-colored path in this partition into a block
rewrite Genome-DIS in the alphabet of blocks
align Genome-DIS against Genome
use the constructed global alignment to decompose Genome into blocks
return block decomposition of Genome
```

Supplementary Note S4: Transforming block-rectangles into block-squares

Two instances of the same block often represent partial copies of this block with vastly different lengths. To visualize the similar region between these block-instances, we represent the aligned regions between them as a *block-square*. Since we only need to identify the approximate start/end positions of the aligned regions, we use the following approach that is faster than edit distance-based alignment.

Given block-instances P and Q of the same block, we find all long shared substrings between them (i.e., shared substrings longer than *minLength*) using the suffix array and the longest common prefix array (default value *minLength*=10). Given a bin size L (default value 100 bp), we say that a long shared substring starting at position x (y) in P (Q) contributes to a bin $floor((x\text{-}y)/L)$ and define the weight of a bin as the total length of long shared substrings contributing to this bin. We further identify a maximum-weight bin b and consider a diagonal $y=b*L+x$ within the block-rectangle. The starting and ending points of this diagonal within the block-rectangle form a block-square.

Supplementary Note S5: GenomeDecoder algorithm for multiple genomes

GenomeDecoder algorithm for *N*=2 genomes. In the case of two genomes, we classify each k-mer in these genomes as either red (if it occurs only in $Genome_1$), blue (if it occurs only in $Genome_2$), or purple (if it occurs in both $Genome_1$ and $Genome_2$).

The labeling of k-mers into red, blue, and purple translates into the labeling of edges in the graph $DB_k(Genome_1 + Genome_2)$, where $Genome_1 + Genome_2$ stands for the union of string-sets $Genome_1$ and $Genome_2$. GenomeDecoder uses the same algorithm to disembroil this graph but modifies edge labels during the disembroiling transformations: the transformation of a collapsible bubble results in a red (blue) path if all edges in this bubble are red (blue) and results in a purple path if either there is a purple edge in this bubble or if it contains both red and blue edges.

The blocks in $Genome_1$-*DIS* and $Genome_2$-*DIS* are generated as described in the case of a single genome. The blocks in $Genome_1$ ($Genome_2$) are generated by aligning them against $Genome_1$-*DIS* ($Genome_2$-*DIS*) using edlib.

GenomeDecoder algorithm for *N*>2 genomes. In the case of more than two genomes, GenomeDecoder uses a multi-step approach for decomposing these genomes into blocks. In each step, it generates the disembroiled genomes for each pair (i^{th} genome, j^{th} genome) using the previously described approach in the case N=2. The pairs of input genomes are disembroiled in the lexicographic order. For example, if N=3, the order of steps is (1st genome, 2nd genome), (1st genome, 3rd genome), and (2nd genome, 3rd genome).

Figure S2 presents the joint block decomposition of human (IGH_{hum}), Bornean orangutan ($IGH_{B.orang}$), and bonobo (IGH_{bonobo}) IGH loci and illustrates large differences in genomic architectures. For example, block K is tandemly repeating 10 times in IGH_{bonobo} and harbor 7 V genes but only 2 times in IGH_{hum} (harbors 3 V genes) and once in $IGH_{B.orang}$ (harbors 3 V genes). Block N is tandemly repeating 6 times in $IGH_{B.orang}$ and harbors 14 V genes and only 1 time in IGH_{hum} (harbors 2 V genes) and 1 time in IGH_{bonobo} (harbors 1 V gene).

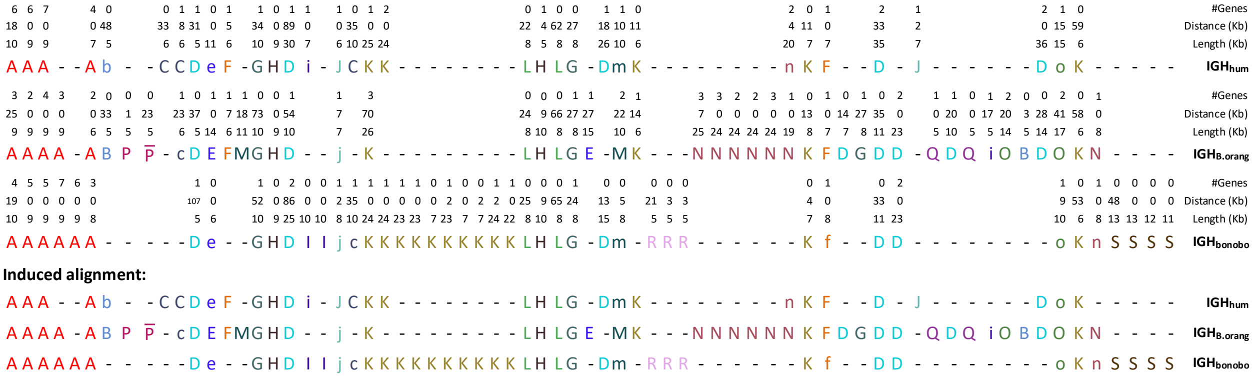

Figure S2. Differential analysis of blocks in IGH$_{hum}$, IGH$_{B.orang}$, and IGH$_{bonobo}$. A reverse-complementary instance of a block is indicated by an overline, e.g., $\overline{P}$ stands for a reverse-complementary instance of a block P. 19 blocks form 33, 47, and 45 block-instances for IGH$_{hum}$, IGH$_{B.orang}$, and IGH$_{bonobo}$, respectively. Only 21 block-instances are shared among all three species, indicating large SVs. Only blocks longer than 5 kb are shown (all blocks longer than 2kb are shown in Supplementary File 3.)

Supplementary Note S6: Comparing genomic architectures of the primate MHC loci

The highly-repetitive Major Histocompatibility Complex (MHC) locus encodes cell surface proteins essential for the vertebrate immune system. We used GenomeDecoder to compare human and Bornean orangutan MHC loci MHC$_{humT2T}$ and MHC$_{B.orang}$. GenomeDecoder with the default parameters generated 11 blocks that form 23 block-instances in MHC$_{humT2T}$ and 31 block-instances in the MHC$_{B.orang}$ (Figure S3). However, visual inspection of the resulting scaled block-plot (Figure S3) revealed that GenomeDecoder with default parameters failed to capture some short blocks. For example, one group of undetected blocks (shown within two red ovals) is seen to the left and below the instances of the orange block F, while another group of undetected blocks (shown within two green ovals) is seen to the left and below the instances of the purple block E. This visual inspection of scaled block-plot is useful because it reveals that the default parameters may miss some highly diverged block-instances.

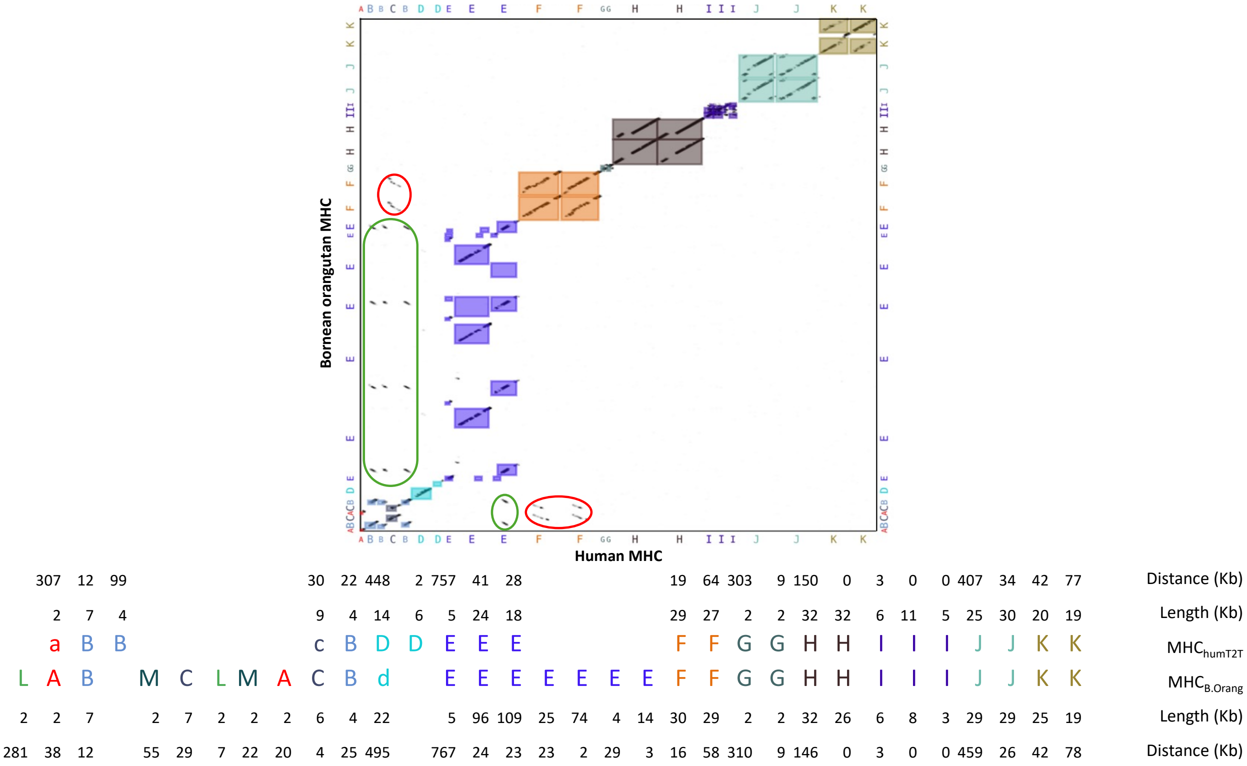

Figure S3. The scaled block-plot (top) and the block decomposition of MHC_{humT2T} and $MHC_{B.orang}$ (bottom) generated using the default parameters of GenomeDecoder (*k*=31, 51, 101, 201, 401, 801, 2001). The bottom Figure shows distances between block-instances (first and sixth lines), lengths of block-instances (second and fifth lines), and the block decompositions (third and fourth lines). A "-" sign before a letter in the scaled block-plot and an overline on a letter in the block representation indicate a reverse complementary block-instance. The scaled block-plot reveals that some (relatively short) block-instances are missing (highlighted using red and green ovals) because the initial *k*-mer size of 31 is too high to identify highly-diverged block-instances.

Reducing the initial *k*-mer size results in a trade-off between the granularity and complexity of the block decomposition. When the divergence between some instances of a block is high, some block-instances are not captured by the initial *k*-mer size (*k*=31) in the default setting. We thus generated the block decomposition with a lower initial *k*-mer size (*k*=21, 25, 31, 51, 101, 201, 401, 801, 2001) that produced 19 duplication blocks with 53 and 59 block-instances in MHC_{humT2T} and $MHC_{B.orang}$, respectively.

This setting revealed a more complex picture of blocks as compared to the default setting, For example, it revealed the block C in Figure S4 with 8 and 10 instances in MHC_{humT2T} and $MHC_{B.orang}$, respectively (the gray block C in Figure S4 corresponds to the orange block F in Figure S3). For the block H (corresponding to the block E in Figure S3), this setting generated one forward block-instance and four reverse block-instances in MHC_{humT2T}, and one forward block-instance and six reverse block-instances in $MHC_{B.orang}$.

This analysis illustrates that varying parameters of GenomeDecoder allows one to identify highly diverged block-instances. For example, the copy number of the block H in Figure S4 is different from the copy number of block E in Figure S3 because the inference of this highly diverged block is parameter-dependent. Since there is no objective function and no ground truth for block decompositions, the utility of these decompositions is defined by downstream applications.

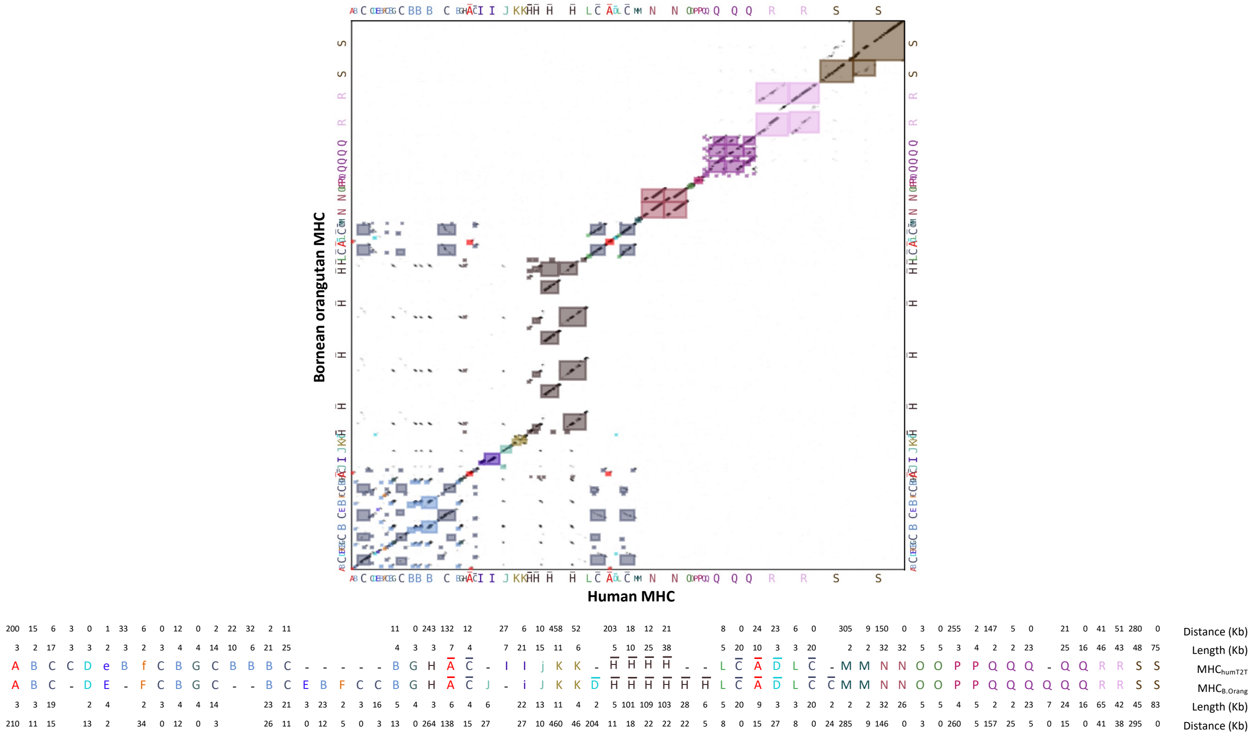

Figure S4. The scaled block-plot (top) and the block decomposition of MHC_{humT2T} and $MHC_{B.orang}$ (bottom) generated using GenomeDecoder with the reduced initial *k*-mer size. The blocks are generated using GenomeDecoder with *k*=21, 25, 31, 51, 101, 201, 401, 801, 2001. Due to limited space, some letters in the axis of the scaled block-plot are overlapping. This setting of GenomeDecoder identified the reverse-complementary instances of block C (gray), missed in Figure S3 and generated more duplication blocks than the default setting in Figure S3 (19 in this Figure and 11 in Figure S3).

Supplementary Note S7: Benchmarking chromosome-scale alignment tools

Evaluating running time of edlib, minimap2, and UniAligner. Even though GenomeDecoder is fast in decomposing long IGH and MHC loci into blocks, it becomes slow in decomposing entire chromosomes into blocks. We thus benchmarked edlib, minimap2, and UniAligner (some of the fastest tools designed for comparing long strings) in aligning the human chromosome X (HUM_X) against its disembroiled version. The benchmarks were conducted on a system with Intel(R) Xeon(R) Platinum 8164 CPU @ 2.00GHz (104 threads across 52 cores).

Edlib (v1.2.7), minimap2 (v2.17), and UniAligner (v0.1) took 48h23m33s, 1m3s (using alignment option asm20), and 48m6s, respectively. We note that it is not a fair comparison: while edlib and UniAligner represent global alignment tools, minimap2 is a local alignment tool that broke the alignment of HUM_X into 1,277 sub-alignments. Because substrings that form these local alignments overlap, there are multiple ways to combine them into a single global alignment. Since this task is beyond the scope of this paper, we decided to use edlib for aligning the original sequence against its disembroiled version in GenomeDecoder.

Evaluating minimap2 alignments. We also considered the possibility of using minimap2 for generating local alignments of each block in the disembroiled chromosome against the original chromosome. This approach is much faster since it substitutes the global alignment of the entire chromosome by a fast procedure that generates local alignments of each (relatively short) block. However, similarly to the conclusion in Bzikadze and Pevzner, 2023, our benchmarking revealed that the accuracy of minimap2 deteriorates in highly-repetitive regions.

To evaluate minimap2 alignments in highly-repetitive regions, we focus on the tandemly repeated block A in IGH_{hum} of length 10,226 bp forming a 4-unit tandem repeat *TR* (Figure 1) and its disembroiled version *TR** in IGH_{hum}-DIS. The edlib-based global alignment of *TR** against *TR* resulted in the block decomposition $A_1A_2A_3A_4$ of *TR* with the following coordinates:

A_1 :18843-28992 (10149 bps), A_2 :28992-38300 (9308 bps), A_3:38300-47710 (9410 bps), A_4 :47710-54689 (6979 bps)

This edlib-based block decomposition (and similar UniAligner-based block decomposition) is consistent with our analysis of the dot-plot of IGH_{hum} against itself (Figure 4). In contrast, as we show below, minimap2-based block decomposition turned out to be inconsistent with the dot-plot of IGH_{hum} against itself.

We aligned *TR** against *TR* using minimap2 with three different parameters:

- Parameter ASM5 (asm-to-ref mapping, for ~0.1% sequence divergence). minimap2 with this parameter generated 4 shortened units of lengths 1515, 1565, 3470, and 2639 bps and thus failed to capture the full-length units of length approximately 10 kb.
- Parameter ASM10 (asm-to-ref mapping, for ~1% sequence divergence). minimap2 with this parameter generated only 3 out of 4 units (of lengths 3110, 3649, and 2650 bps) and thus failed to capture the full-length units.
- Parameter ASM20 (asm-to-ref mapping, for ~5% sequence divergence). minimap2 with this parameter generated only 2 out of 4 units (of lengths 7396, and 2650 bps) and thus failed to capture the full-length units of length approximately 10 kb.

Based on these tests, we concluded that minimap2 is not well suited for constructing alignments in highly-repetitive regions.

Evaluating edlib alignments. Even though edlib and UniAligner usually produce very similar alignments, edlib sometimes produces biologically inadequate alignments in HRRs (Figure S5). Subsection "Block decomposition contributes to gene identification in IGH loci" in the Results section analyzes alignments of block A (shown in Figure 1) that forms tandem repeats in various ape genomes. Alignments of some gene-centered windows reveal that an annotated IG gene in its human block-instance (denoted $A1_{hum}$) was disrupted in some A-blocks. Figure S5 shows alignments of the gene IGHD1-20 in $A1_{hum}$ against $A4_{hum}$ has a very low window PI of 36%, suggesting that IGHD1-20 was disrupted in $A4_{hum}$. It turned out that this alignment represents an artifact of the edlib tool since the region where IGHD1-20 occurred in $A1_{hum}$ was deleted in $A4_{hum}$ (Figure 1 illustrates that $A4_{hum}$ has length 6 kb

while A1hum has length 10 kb). UniAligner (Bzikadze and Pevzner, 2023) generates a more realistic alignment of this region that shows the deletion of the entire window.

A Using Edlib

```
Gene IGHD3-22 in A1_hum vs A4_hum (IGHD3-3)

(gene PI=78%, window PI=89%, heptamer mutations: (0/0), nonamer mutations: (2/1)

AGGCATGAGTTTTTGATGGACTCTGTGACACTGTGG-TAGTAATAACCACTACTATCATAGTAATACCACAGTGACACAGACCTCACTTCAAACCTACCGC

|||.|||.||||||||||||||||||||||||||||-||-|||||||||||.|.|..||.|||||||||||||||||||||||||||..||||||||||||

AGGGATGGGTTTTTGATGGACTCTGTGACACTGTGGGTA-TAATAACCACTCCAAAAATCGTAATACCACAGTGACACAGACCTCACCCCAAACCTACCGC
```

B Using Edlib

```
Gene IGHD1-20 in A1_hum vs A4_hum

(gene PI=23%, window PI=36%, heptamer mutations: (5/4), nonamer mutations: (7/5)

TGCTGCCAGGAGTCAGTTTTGGACACAGTTTTTCTCACGGTGGTCGTTCCAGTTATACCCACTGTGACTCGGGGCTGTTCAGAATCCGACCAGGCGCCCT

-|-|-|--||--|||.|---|-||.|||||------||--|---------|-|----||-|--|---|--|||-||---|-----|-|-----------|

-G-T-C--GG--TCACT---G-ACTCAGTT------AC--T---------A-T----CC-A--G---C--GGG-CT---C-----C-G-----------T
```

C Using UniAligner

```
Gene IGHD1-20 in A1_hum vs A4_hum

(gene PI=0%, window PI=0%, heptamer mutations: (7/7), nonamer mutations: (9/9)

TGCTGCCAGGAGTCAGTTTTGGACACAGTTTTTCTCACGGTGGTCGTTCCAGTTATACCCACTGTGACTCGGGGCTGTTCAGAATCCGACCAGGCGCCCT

-----------------------------------------------------------------------------------------------------

-----------------------------------------------------------------------------------------------------
```

Figure S5. Alignments of genes IGHD3-22 and IGHD1-20 in $A1_{hum}$ against $A4_{hum}$. A. Edlib alignment of IGHD3-22 against $A4_{hum}$. **B.** Edlib alignment of IGHD1-20 against $A4_{hum}$. **C.** UniAligner alignment of IGHD1-20 against $A4_{hum}$.

Supplementary Note S8: Benchmarking halSynteny

Benchmarking halSynteny on simulated DNA sequences. The halSynteny tool (Krasheninnikova et al., 2020) generates blocks by constructing a chain of non-overlapping local alignments between a substring A in one genome and a substring B in another genome and forming a block-rectangle with sides A and B in the 2-D dot-plot (Figure S6). Even though different block-rectangles do not overlap in 2-D, their projections on each genome may overlap, making it difficult to infer a representation of each genome in the alphabet of non-overlapping blocks. In contrast, GenomeDecoder represents each genome in the alphabet of non-overlapping blocks. Each pair of instances of the same block also forms a block-rectangle, but in difference from halSynteny, projections of every two block-rectangles are either identical (if they are formed by the same block) or do not overlap in 1-D.

Since halSynteny uses various parameters for inferring blocks, we attempted to find parameters that generate non-overlapping (or weakly overlapping) blocks and thus enable comparison with GenomeDecoder. Below we use simulated genomes that illustrate the differences in block generation between these two tools.

We benchmarked GenomeDecoder and halSynteny on two simulated sequences S_1 and S_2 with just five predefined blocks: $S_1 = X_1$AAABDCCCBX_2 and $S_2 = X_3$AAAADECCBX_4. Each of blocks A, B, C, D and E is a 3kb long randomly generated nucleotide sequence. X_1, X_2, X_3, and X_4 are distinct 2kb long randomly generated sequences at the starts and ends of S_1 and S_2. The correct set of block-rectangles for these genomes consists of 21 block-rectangles (3x4 rectangles for A, 2x1 rectangles for B, 3x2 rectangles for C, 1 rectangle for D, and 0 rectangles for E).

Figure S6 illustrates that GenomeDecoder generated all 21 correct block-rectangles (for K=2001). halSynteny generated block-rectangles with overlapping projections (for all values of parameters we explored).

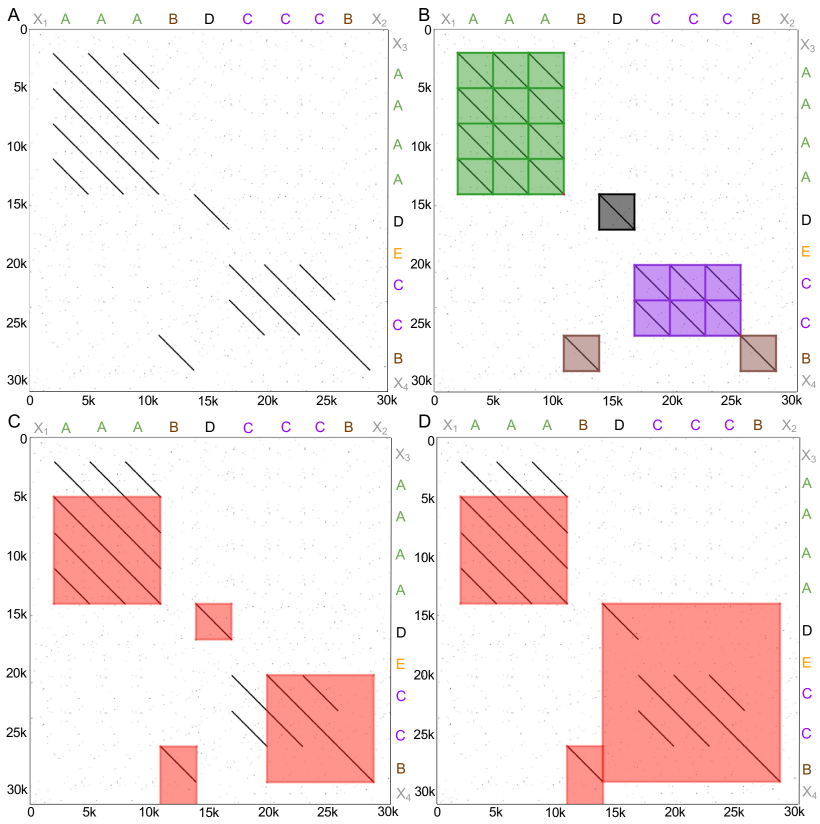

Figure S6. Genomic dot-plot of simulated sequences S_1=X_1AAABDCCCBX_2 and S_2=X_3AAAADECCBX_4 (A) and their block decompositions by GenomeDecoder (B) and halSynteny (C and D). The letters at the top and right of each plot show the block decomposition of S_1 (x-axis) and S_2 (y-axis). **A.** Dot-plot of S_1 and S_2. **B.** The block-rectangles generated by GenomeDecoder with the default parameters. **C.** The block-rectangles generated by halSynteny with parameters *minBlockSize*=500bp, and *maxAnchorDistance*=2 kb. halSynteny generated just 4 block-rectangles, moreover, it excluded the first block-instance of A in S_2, and the first block-instance of C in S_1 **D.** The block-rectangles generated by halSynteny with parameters *minBlockSize*=500bp, and *maxAnchorDistance*=3 kb. halSynteny generated just 3 block-rectangles that excluded the first block-instance of A in S_2.

Benchmarking halSynteny on primate IGH loci. We compared the synteny blocks generated by halSynteny and GenomeDecoder for IGH_{hum} and $IGH_{B.orang}$ loci. While both GenomeDecoder and halSynteny produced blocks that were consistent with the long local alignments between these loci, there were important differences in the boundaries of the generated block-instances. Below we focus on some blocks generated by GenomeDecoder to illustrate why non-overlapping block representation by GenomeDecoder (as opposed to the overlapping representation by halSynteny) is instrumental in understanding genomic architecture. Specifically, we focus on blocks A and H that form tandem repeats (Figure 6B). The tandem repeat A harbors the diversity (D) genes and occurs five times in orangutan (spanning positions from 25 kb to 68 kb) and four times in human IGH locus (spanning positions from 18 kb to 53 kb) (Figure 6B). In contrast to GenomeDecoder, halSynteny failed to reveal the structure of this tandem repeat. The tandem repeat H occurs six times in $IGH_{B.orang}$ (spanning positions from 824 kb to 958 kb) and only once in IGH_{hum} (spanning positions from 757 kb to 769 kb). GenomeDecoder inferred all the six instances of the block H while halSynteny reported only four of them.

Supplementary Note S9: Benchmarking Sibelia

Sibelia iteratively constructs a series of de Bruijn graphs with increasing *k*-mer sizes that end with the largest *K*-mer size (default value *K*=5 kb). Below, we focus on Sibelia-generated blocks in IGH_{hum} inferred using its default *loose* setting as it is supposed to generate fewer but longer blocks. Like GenomeDecoder, Sibelia uses RepeatMasker to mask common repeats. We applied Sibelia for generating blocks in the IGH loci for a variety of parameters, including setting *K*=2 kb (as we did for GenomeDecoder), but these parameters generated suboptimal results as compared to

Sibelia's default parameters. We did not benchmark the Sibelia-Z tool (Minkin and Medvedev, 2020) since it was primarily developed for a slightly different goal of multiple genome-scale alignment.

Comparing block decompositions generated by Sibelia and GenomeDecoder. Figure 5 compares 7 blocks generated by Sibelia (A, B, F, E, K, L, and N) with 14 blocks generated by GenomeDecoder that are shown in Figure 1. Below, we analyze some differences between these decompositions of the IGH_{hum} locus:

- Both Sibelia and GenomeDecoder identified the tandem repeat AAAA of multiplicity 4 but assigned different lengths to its block-instances/ while Sibelia assigned a length of ≈6 kb to all four instances of this block, GenomeDecoder assigned lengths 10, 9, 9, and 6 kb to these blocks. Figure S7A and S7B suggest that GenomeDecoder generated a more adequate representation of this tandem repeat.
- Sibelia missed rather long blocks C and D of lengths 6 kb and 8 kb, respectively. Figure S7C and S7D show the dot-plots of the regions corresponding to two pairs of instances of these blocks respectively and illustrate that both instances of C and both instances of D are well aligned. We hypothesize that Sibelia missed these blocks because their inference required the processing of some complex bubbles in the de Bruijn graphs.
- GenomeDecoder generated block E of length ≈25 kb, while Sibelia partitioned this block into two blocks, E_1 and E_2, of lengths approximately 13 and 12 kb. These two representations are equivalent, with only cosmetic differences. However, GenomeDecoder found partial occurrences of shorter segments of block E (of length 3 kb and 7 kb) that Sibelia missed (Figure 5).

Time and memory usage of GenomeDecoder and Sibelia. Since our analysis revealed that Sibelia generated the most adequate block decompositions, we only benchmark the time and memory usage of GenomeDecoder and Sibelia (Table S1). Since GenomeDecoder has a more involved graph simplification algorithm than Sibelia, it has a significantly higher runtime and memory usage. However, the increased runtime and memory usage of GenomeDecoder represents a reasonable trade-off since, as our benchmarking illustrated, it generates more adequate block decompositions.

Table S1. Time and memory usage of GenomeDecoder and Sibelia. All runs used an Intel(R) Xeon(R) Platinum 8164 CPU @ 2.00GHz machine with 104 CPUs using default parameters.

Genome	Total length (kb)	**GenomeDecoder**				**Sibelia**	
		Running time (disembroiling)	Memory usage (disembroiling)	Running time (alignment)	Memory usage (alignment)	Running time	Memory usage
IGH_{hum}	1,017	37s	69 MB	21s	207 MB	5s	35 MB
$IGH_{B.orang}$	1,416	96s	100 MB	65s	288 MB	8s	47 MB
HUM_{20}	64,444	29m2s	1.0 GB	2h31m24s	7 GB	7m27s	1.5 GB
HUM_{X}	156,041	12h56m54s	2.3 GB	48h23m33s	16 GB	20m11s	3.7 GB
IGH_{hum} vs. IGH_{humT2T}	1,017 + 1,161	144s	130 MB	102s	231 MB	15s	69 MB
IGH_{hum} vs. $IGH_{B.orang}$	1,017 + 1,416	9m46s	244 MB	2m0s	291 MB	23s	82 MB
HUM_{20} vs. $HUMT2T_{20}$	64,444 + 66,210	7h23m5s	3.7 GB	3d0h16m58s	7.9 GB	16m38s	3.1 GB
HUM_{X} vs. $HUMT2T_{X}$	156,041 + 154,260	1d18h23m1s	7.2 GB	16d21h55m11s	18.3 GB	45m45s	8.4 GB
IGH_{hum} vs. $IGH_{B.orang}$ vs. IGH_{bonobo}	1,017 + 1,416 + 1,183	35m47s	244 MB	6m53s	304 MB	41s	162 MB

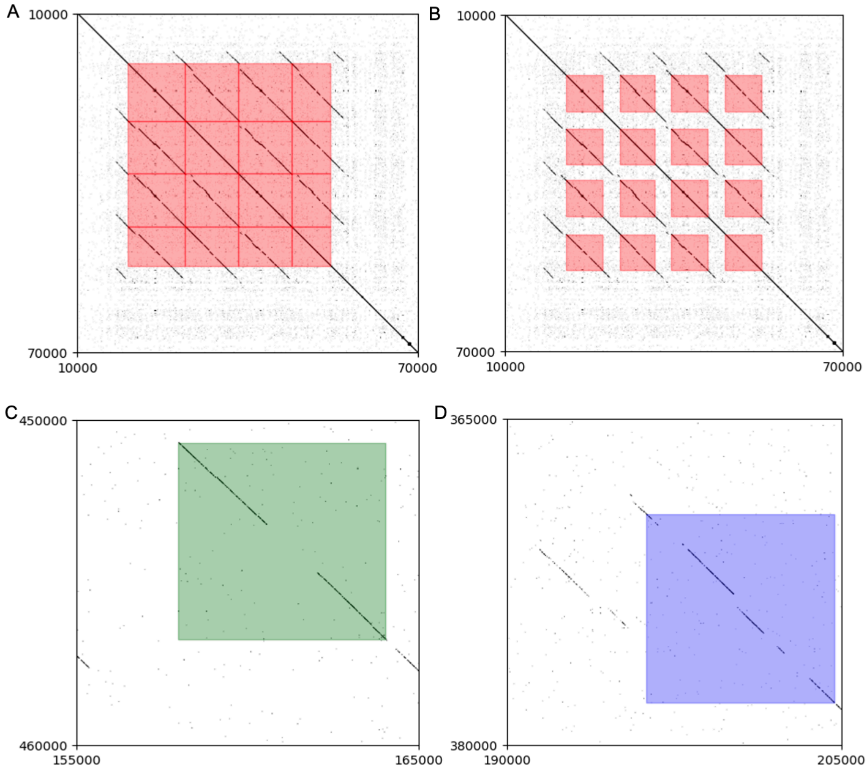

Figure S7. Comparing block decompositions generated by GenomeDecoder and Sibelia for the IGH$_{hum}$ locus. A-B. Region of block A identified by GenomeDecoder (subplot A) and Sibelia (subplot B). Both GenomeDecoder and Sibelia identified the tandem repeat AAAA of multiplicity 4 but assigned different lengths to its instances. **C-D.** GenomeDecoder identified block C (subplot C) with two block-instances of lengths 6053 and 6036 bp, and block D (subplot D) with two instances of lengths 8437 and 8662 bp. UniAligner aligned block-instances of C with 5439/320/277/294 matches/mismatches/insertions/deletions and block-instances of D with 7094/500/1068/843 matches/mismatches/insertions/deletions.

Supplementary Note S10: Benchmarking SDquest

With default parameters, SDquest decomposed IGH$_{hum}$ into 567 block-instances of 104 non-overlapping blocks varying in length from 104 to 25042 bp. These blocks have been provided in Supplementary File 4 and visualized as a block-plot in Supplementary File 5. Out of these, there were 78 block-instances of 32 blocks longer than 2kb that we consider below (GenomeDecoder has the default parameter K=2 kb). These blocks covered in total ≈300 kb of IGH$_{hum}$.

The repeating blocks generated by GenomeDecoder covered regions with approximately the same total length but with only 37 block-instances of 14 blocks. The manual analysis revealed that SDquest often breaks the larger blocks into smaller ones, making their further analysis rather complicated. For example, given a segment AxByAzB, (x, y, and z stand for short non-repetitive sequences), SDquest decomposes it as ABAB (because blocks A and B are separated by non-repetitive sequences) while GenomeDecoder forms a block C=AxB and decomposes this segment as CC. This is not an error of SDquest but merely an example of over-fragmenting the block decomposition. While this simple over-fragmentation can be easily addressed by post-processing SDquest output, it is not clear how to address more complex over-fragmentation artifacts.

The authors of SDquest noticed this over-fragmentation and made the first step towards enlarging the SDquest blocks by combining them into SD-units (Pu et al., 2018). However, this post-processing has addressed some but not all over-fragmentation artifacts since it gives rise to 60 units (termed as SD-units by Pu et al., 2018) with total number of instances (sum of multiplicities) equal to 342. These units are shorter (have lengths ranging from 116 to 8095 bp) and have higher multiplicities (multiplicities ranging from 2 to 28) than the GenomeDecoder blocks for IGH$_{hum}$, indicating over fragmentation. To further analyze the over-fragmented blocks generated by SDquest, we focused on three largest

duplicated blocks in IGH_{hum} shown in Figure 1: block A (largest block-instance has length 10 kb), block E (largest block-instance has length 26 kb), and block K (largest block-instance has length 45 kb). The 4-unit tandem repeat AAAA (18kbp - 56kbp) forms the IGHD locus and has four ≈10kbp long block-instances as suggested by the genomic dotplot and the detailed analysis of the human IGHD locus in Safonova and Pevzner, 2020. However, the blocks produced by SDquest split the IGHD locus into 38 block-instances of 10 blocks.

```
ABCDEFGHIJABDEFGHIJABCDEFGHIJABDEFGHIJ
```

This is a correct but over-fragmented block decomposition that makes further analysis unnecessary complex. The red, blue, green, and purple segments in this decomposition correspond to a single block in the GenomeDecoder decomposition that forms a 4-unit tandem repeats.

```
ABCDEFGHIJ
AB-DEFGHIJ
ABCDEFGHIJ
AB-DEFGHIJ
```

GenomeDecoder "embroils" the differences between 4 block-instances of A in the course of the iterative embroilment procedure. Thus, the key difference between SDquest and GenomeDecoder/Sibelia is that SDquest implicitly performs a single iteration of block generation while GenomeDecoder/Sibelia perform multiple iterations to avoid over-fragmentation. While in the simple case described above, it is easy to post-process SDquest output to avoid over-fragmentation, this post-processing becomes non-trivial in more complex cases.

To address over-fragmentation of SDquest blocks, we have developed an SDquest+GenomeDecoder post-processing pipeline that takes a given SDquest block decomposition of a genome and generates the simulated version of this genome with the same block decomposition. Afterwards, it submits the resulting simulated genome to GenomeDecoder for generating enlarged blocks.

Given a block decomposition of a genome, we generate the simulated genome as follows:

1. Generate a random sequence rand(X) for each block X (including non-repetitive blocks). The length of rand(X) is set as the mean length of all block-instances of the block X;
2. Substitute each instance of each block X in the resulting block decomposition by the sequence rand(X) so that all block-instances of the same block are identical;

We run GenomeDecoder on the resulting simulated genome to alleviate over-fragmentation in the original block decomposition. Applying this approach to SDquest blocks results in a pipeline that we refer to as SDquest+GenomeDecoder.

SDquest+GenomeDecoder generates a less fragmented decomposition of the IGH locus with 51 block-instances of 16 blocks. Figure S8 illustrates that this decomposition is quite similar to the GenomeDecoder decomposition with 37 block-instances of 14 blocks shown in Figure 1.

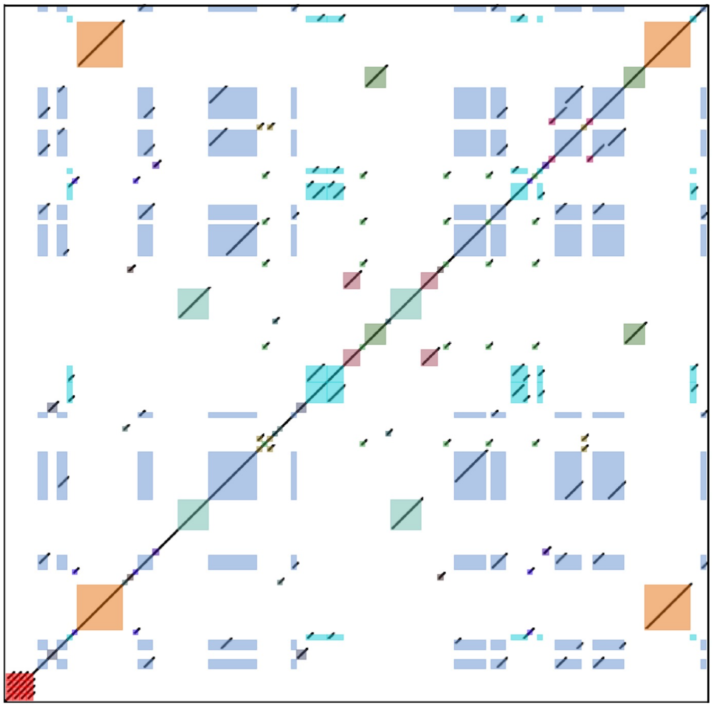

Figure S8. The block-plot of the block decomposition generated by SDquest+GenomeDecoder. SDquest decomposes IGH_{hum} into 567 block-instances of 104 blocks. This over-fragmented decomposition makes comparison of genomic architectures difficult. SDquest+GenomeDecoder simplifies the decomposition produced by SDquest after substituting the block-instances by random sequences and running GenomeDecoder on the resulting simulated sequence. For example, the first block in the new decomposition (red) is a tandem repeat of four block-instances, the same as block A in the decomposition produced by running GenomeDecoder alone.

Supplementary Note S11: Diversifying selection in immunoglobulin loci

Recent studies have revealed that the success of an antibody response to a specific antigen often depends on a specific IG gene. For instance, the response to flu is primarily mediated by antibodies derived from the IGHV1-69 gene (Avnir et al., 2016), whereas the response to *Plasmodium falciparum* is almost exclusively mediated by antibodies derived from the IGHV3-33 gene (Tan et al., 2018). Thus, the *usage* of such disease-specific IG genes (i.e., the fraction of antibodies derived from them among all antibodies in an antibody repertoire) represents an important evolutionary-optimized feature.

Indeed, the increased usage of a disease-specific IG gene increases the success rate of the antibody response since it reduces the expected number of rounds of somatic hypermutations needed to optimize the naïve antibodies derived from this gene. For example, high usage of anti-malaria IGHV3-33 is more crucial for survival in sub-Saharan Africa than in the US. However, increased usage of IGHV3-33 inevitably reduces usage of other IG genes that contribute to immune response against other pathogens. Thus, the IG gene usage must be optimized for each location based on the spectrum of pathogens prevalent there. Moreover, mammals are constantly affected by newly emerged pathogens, which may need specific IG genes for mounting a successful antibody response. It is thus beneficial to have a population with varied IG gene usage so that individuals with increased usage of disease-specific IG genes are better prepared for new infections. This argument falls under the *diversifying* (*disruptive*) selection mode of evolution that happens when extreme phenotypes have a fitness advantage over average phenotypes (Martin et al., 2012, Kondrashov, 2012).

A duplication of a block that harbors a disease-specific IG gene increases its usage in the antibody repertoire. For example, Figure 6A shows the tandem duplication K → KKK in IGH_{hum} compared to IGH_{humT2T}, while Figure 6B shows the tandem duplication H → HHHHHH in $IGH_{B.orang}$ compared to IGH_{hum}. Such duplications tend to proportionally increase the usage of genes located in the duplicated blocks; for instance, the usage of genes in the block H in Bornean orangutan is expected to increase six-fold as compared to human (Stranger et al., 2007, Rodriguez,

2023). We hypothesize that the highly increased rate of segmental/tandem duplications in the IG loci is an evolutionary mechanism ensuring that the population includes individuals capable of mounting antibody response against location-specific and new pathogens. This mechanism does not necessarily optimize the survival chances of a specific individual (since this individual may have a suboptimal IG gene usage with respect to a newly emerged pathogen) but instead optimizes the survival chances of the population by ensuring that at least some individuals can mount successful antibody responses. Moreover, duplicating a block-instance allows IG genes in this block-instance to continue mounting response to existing infections while a new copy is free to evolve for optimizing response to a new infection.

The Human Pangenome Reference Consortium is now generating complete genomes across diverse human sub-populations. Analysis of the IGH loci in these genomes may provide a way to confirm or reject the stated hypothesis. For example, if sub-Saharan genomes have an increased number of copies of the block E from Figure 6A as compared to European genomes it may indicate the increased usage of the anti-malaria gene IGHV3-33 gene that resides within this block.

Supplementary Note S12: Parameters of GenomeDecoder

Input Genomes. The "-g" parameter in GenomeDecoder specifies the input genome file in the FASTA format. Each genome file should contain a single string (in the case of a multi-chromosomal genome, all chromosomes should be concatenated in a single string using "N" as a separator). To generate the joint block decomposition of *m* genomes, the "-g" parameter should be specified *m* times. The genomes should be repeat-masked by RepeatMasker before inputting into GenomeDecoder to ensure proper functionality.

List of *k*-mer sizes for the iterative genome disembroiling procedure. The default list of *k*-mer sizes is (31, 51, 101, 201, 401, 801, 1601, 2001). GenomeDecoder ends genome disembroiling at the default *K*-mer size *K*=2001 (the minimum block size). We used the default list of *k*-mer sizes for all results in this manuscript with the exception of analysis of the MHC loci in Supplementary Note S6 where we generated block decomposition with both the default and modified list of *k*-mer sizes. Users can set the list of *k*-mer sizes using the parameter "-k" in GenomeDecoder.

Similarity threshold parameters. Given a percent identity *sim*, two strings are classified as *sim*-similar if the percent identity between them (as defined by the edlib alignment) exceeds a threshold *sim*.

A simple bubble in the graph $DB_k(Genome)$ is classified as collapsible if its edges spell *sim*-similar sequences with respect to the parameter *sim.* GenomeDecoder uses different similarity thresholds for simple bubble collapsing at various iterations: the threshold $sim=sim_{strong}$ (default value *sim*=90%) at the initial iteration and a user-controlled smaller threshold $sim=PI_{simple}$ (default value *sim*=0%) at the follow-up iterations. Our analysis revealed that the lower similarity threshold at the follow-up iterations addresses the high variation of block instances in complex genomic regions. Users can set the PI_{simple} threshold using the parameter "-s" in GenomeDecoder.

A complex bubble in the graph $DB_k(Genome)$ is classified as collapsible if its two paths spell *weakly-similar* sequences with respect to the parameter $sim=sim_{weak}$ (default value *sim*=65%). This user-controlled threshold can be set using the "-c" parameter in GenomeDecoder.

Repetition parameter *Repeat* for generating block decomposition of multiple genomes. GenomeDecoder uses the following multi-step approach for generating block decomposition of *N* genomes for *N*>2. In each iteration, it generates the disembroiled genomes for each pair (i^{th} genome, j^{th} genome) of genomes. The pairs of input genomes are disembroiled in the lexicographic order, for example, if *N*=3, the order is (1st genome, 2nd genome), (1st genome, 3rd genome), (2nd genome, 3rd genome). This process is repeated *Repeat* times (default value *Repeat*=5). Users can adjust the *Repeat* parameter with the '-i' parameter in GenomeDecoder.

References

Avnir, Y., Watson, C., Glanville, J. *et al.* IGHV1-69 polymorphism modulates anti-influenza antibody repertoires, correlates with IGHV utilization shifts and varies by ethnicity. *Sci Rep* 6, 20842 (2016).

Bzikadze AV, Pevzner PA. UniAligner: a parameter-free framework for fast sequence alignment. *Nat Methods*. 2023, 20:1346-1354.

Kondrashov FA. Gene duplication as a mechanism of genomic adaptation to a changing environment. *Proceedings of the Royal Society* (*Biological Sciences*) 2012; 279, 5048-57.

Krasheninnikova, K., Diekhans, M, Armstrong, J., Dievskii, A., Paten, B, O'Brien, S. halSynteny: a fast, easy-to-use conserved synteny block construction method for multiple whole-genome alignments, *GigaScience*, 2020, 9, giaa047.

Martin RA, Pfennig DW. Widespread disruptive selection in the wild is associated with intense resource competition. *BMC Evol Biol*. 2012 2;12:136.

Minkin, I., Medvedev, P. Scalable multiple whole-genome alignment and locally collinear block construction with SibeliaZ. *Nature Communications*, 2020, 6327.

Pu L, Lin Y, Pevzner PA. Detection and analysis of ancient segmental duplications in mammalian genomes. *Genome Research*. 2018; 28:901-9.

Rodriguez, O.L., Safonova, Y., Silver, C.A. et al. Genetic variation in the immunoglobulin heavy chain locus shapes the human antibody repertoire. *Nature Communications* 2023,14, 4419 .

Safonova, Y, and Pevzner, P.A. "V (DD) J recombination is an important and evolutionarily conserved mechanism for generating antibodies with unusually long CDR3s." *Genome Research* 30.11 (2020): 1547-1558.

Sosic, M, Sikic, M. Edlib: a C/C ++ library for fast, exact sequence alignment using edit distance. *Bioinformatics*, 33, 1394-95, 2017.

Stranger BE, Forrest MS, Dunning M, Ingle CE, Beazley C, Thorne N, Redon R, Bird CP, de Grassi A, Lee C, Tyler-Smith C, Carter N, Scherer SW, Tavaré S, Deloukas P, Hurles ME, Dermitzakis ET. Relative impact of nucleotide and copy number variation on gene expression phenotypes. *Science*. 2007; 315:848-53.

Tan J, Sack BK, Oyen D, Zenklusen I, Piccoli L, Barbieri S, et al. A public antibody lineage that potently inhibits malaria infection through dual binding to the circumsporozoite protein. *Nature Medicine*. 2018;24:401–7.
